# Supplementary material for: Clinical subphenotypes in COVID-19: derivation, validation, prediction, temporal patterns, and interaction with social determinants of health
Source: NPJ Digit Med. 2021 Jul 14;4:110. doi: 10.1038/s41746-021-00481-w (PMC8280198; doi:10.1038/s41746-021-00481-w)
Supplement: Supplementary file 1 — Supplementary Information [file 41746_2021_481_MOESM1_ESM.pdf]

Supplementary information to:

Clinical subphenotypes in COVID-19: derivation, validation, prediction, temporal patterns, and interaction with social determinants of health

Authors: Chang Su[1], Yongkang Zhang[1], James H Flory[2], Mark G. Weiner[1], Rainu Kaushal[1,3,4*], Edward J. Schenck[3,5*], Fei Wang[1*]

[1]Department of Population Health Sciences, Weill Cornell Medicine, New York, NY, USA;

[2]Memorial Sloan-Kettering Cancer Center, New York, NY, USA;

[3]New York-Presbyterian Hospital, Weill Cornell Medicine, New York, NY, USA;

[4]Department of Medicine, Weill Cornell Medical College, New York, NY, USA;

[5]Division of Pulmonary & Critical Care Medicine, Joan and Sanford I. Weill Department of Medicine, Weill Cornell Medicine, New York, NY, USA

*Corresponding authors

Corresponding Authors:

Rainu Kaushal,
515 E 71st St, New York, NY 10021. USA
rak2007@med.cornell.edu

Edward J. Schenck,
1300 York Ave. Box 96 New York City. NY 10065. USA
ejs9005@med.cornell.edu

Fei Wang,
425 E 61 St. New York City. NY 10065. USA
few2001@med.cornell.edu

Supplementary Note 1. COVID-19 definition

COVID-19 were defined as either having at least one positive laboratory test result or at least one ICD-10 diagnosis code available in INSIGHT database, including:

- LOINC code 94500-6, SARS coronavirus 2 RNA [Presence] in Respiratory specimen by NAA with probe detection;
- LOINC code 94309-2, SARS coronavirus 2 RNA [Presence] in Unspecified specimen by NAA with probe detection;
- LOINC code 94507-1, SARS coronavirus 2 IgG Ab [Presence] in Serum or Plasma by Rapid immunoassay;
- LOINC code 94508-9, SARS coronavirus 2 IgM Ab [Presence] in Serum or Plasma by Rapid immunoassay;
- LOINC code 94306-8, SARS coronavirus 2 RNA panel - Unspecified specimen by NAA with probe detection;
- ICD-10 code B34.2, Coronavirus infection, unspecified site;
- ICD-10 code B97.21, SARS-associated coronavirus as the cause of diseases classified elsewhere;
- ICD-10 code B97.29, Other coronavirus as the cause of diseases classified elsewhere;
- ICD-10 code J12.81, Pneumonia due to SARS-associated coronavirus;
- ICD-10 code U07.1, COVID-19, virus identified.

Supplementary Note 2. Details of data preparation

Data distributions and missing values of the studied cohorts were assessed (see Supplementary Tables 2 and 3). 7 out of 30 candidate clinical variables were excluded for cluster analysis for subphenotype derivation, due to high missing rate (missing more than 70% values). The 7 high-

missing variables were included in sensitivity analysis. Details of usage of the clinical variables were presented in the Supplementary Table 4.

Logarithmic transformation was applied to the non-normal distributed variables (see Supplementary Table 4). In order to eliminate the effects of value magnitude, all variables were scaled based on z-score. More specifically, in the development cohort, z-score is calculated by

$$x' = \frac{x - \mu}{\sigma}$$

where x is the raw value, and μ and σ are the mean and standard deviation of the samples. For each validation cohort, z-score is calculated by:

$$x' = \frac{x - \mu_{dev}}{\sigma_{dev}}$$

where μ_{dev} and σ_{dev} are the mean and standard deviation of the samples of the development cohort.

To address missing values, we used the K-nearest neighbors (KNN) imputation algorithm, which typically selects top K samples most similar to the sample of interest to impute missing values[1]. According to the previous study. we set K=10 in this analysis. Of note, the KNN imputation model was fitted in development cohorts, and then used to impute missing values in development, internal validation, and external validation cohorts.

Supplementary Note 3. Agglomerative hierarchical clustering

In this study, agglomerative hierarchical clustering analysis was used to derive subphenotypes. On one hand, unlike other clustering methods like k-means clustering, agglomerative hierarchical clustering is usually robust as it’s not sensitive to data distribution (e.g., k-means requires a sphere-like distribution of the data) and doesn’t need an initialization procedure that may

incorporate uncertainty. On the other hand, agglomerative hierarchical clustering typically produces a tree diagram known as dendrogram, which visually illustrates how the data points are agglomerated together in a hierarchical manner and distances between the clusters at different layers in the hierarchy, providing visible guidance in determining the optimal cluster number. For example in Supplementary Fig. 3a, each data point (i.e., patient) is considered as a separate cluster at the beginning. Then, at each step, the two clusters that are most similar are joined into a single new cluster. The vertical axis of the dendrogram represents the distance or dissimilarity between clusters. High inter-cluster distance indicates a clear cluster structure of the data. Here, we performed agglomerative hierarchical clustering with Euclidean distance calculation based on clinical variable of the patients. Ward linkage criterion[2] was used to construct the hierarchy.

'NbClust'[3] is a famous R package that was developed to assist a clustering method to determine the optimal cluster number of the data. In this study, we used 21 indices provided by 'NbClust' to evaluate cluster structure of the agglomerative hierarchical clustering model with Ward criterion. The optimal cluster number was determined by the optimal value of each index. The used indices included: KL index[4], CH index[5], Hartigan index[6], CCC index[7], Scott index[8], Marriot index[9], TrCovW index[10], TraceW index[10], Friedman index[11], Rubin index[11], Cindex[12], DB index[13], Silhouette index[14], Duda index[15], Pseudot2 index[15], Beale index[16], Ratkowsky index[17], Ball index[18], Ptbiserial index[19], Frey index[20], McClain index[21], Dunn index[22], Hubert index[23], SDindex[24], Dindex[25], and SDbw index[26]. More specifically, we set the cluster number k ranging from 2 to 8 and perform the clustering algorithm. Based on cluster structure identified given a specific k, the 21 indices are calculated. Each index measures how good the cluster structure is. For example, the maximum value of KL index indicates the optimal cluster structure given a specific cluster number. In this way, each of the 21 indices will suggest an optimal cluster number. Finally, the overall optimal cluster number can be determined by majority voting.

Supplementary Note 4. Gaussian mixture model

Another clustering algorithm, Gaussian mixture model (GMM)[27], was used in sensitivity analysis to re-derive subphenotypes in the development cohort. Compared to agglomerative hierarchical clustering that is based on distance among data points, the GMM is a probabilistic model that assumes all the data points are generated from a mixture of a finite number of Gaussian distributions with unknown parameters. Typically, Akaike information criterion (AIC), Bayesian information criterion (BIC), and median probability of group membership are used to measure cluster structure of GMM to determine the optimal cluster number. In this analysis, we comprehensively considered these three measures to determine cluster number in sensitivity analysis.

Supplementary Note 5. Subphenotype visualization

Uniform manifold approximation and projection (UMAP) plots

UMAP is a novel dimension reduction technique that are widely used for data visualization[28]. Via a non-linear transformation, UMAP is able to project the high-dimensional data into a low-dimensional space, such that close data points in the original space will be close in the low-dimensional space, and vice versa. Compared to other dimension reduction methods, such as t-distributed stochastic neighbor embedding (t-SNE), UMAP is faster and better preserves the data's global structure. Therefore, we performed UMAP on the clinical variables to visualize patients in the 2-D space. Patients' subphenotype membership were colored in the UMAP plots. In this study, UMAP plots were created by 'umap-learn' package in Python.

Chord diagram

Chord diagram plots were generated to visualize patterns of abnormal clinical variables and chronic comorbidities by subphenotypes, based on 'Circlize' package[29] in R.

Subphenotype vs. abnormal clinical variables. First of all, we grouped the clinical variables into the following categories:

- **Inflammatory markers**: C-reactive protein (CRP), erythrocyte sedimentation rate (ESR), interleukin 6 (IL-6), procalcitonin, bands, lactate dehydrogenase (LDH), lymphocyte count, neutrophil count, white blood cell count, albumin, and ferritin.
- **Hepatic markers**: albumin, ferritin, alanine aminotransferase (ALT), aspartate aminotransferase (AST), and bilirubin.
- **Cardiovascular markers**: creatine kinase (CK), lactate, troponin I, and troponin T.
- **Renal makers**: bicarbonate, blood urea nitrogen (BUN), creatinine, chloride, and sodium.
- **Hematologic markers**: d-dimer, hemoglobin, platelet count, prothrombin time, red blood cell distribution width (RDW), and glucose.

More specifically, for each subphenotype, if median of a clinical variable (e.g., CRP) is more abnormal than the median for the development cohort, we added a ribbon with one unit width between the subphenotype and the specific variable group (e.g., inflammatory markers). In this way, a broader ribbon between a subphenotype and a variable group means the subphenotype has more abnormal varaibles in this group compared to others.

Subphenotypes vs. comorbidities. We assessed nine chronic comorbidities, including hypertension, diabetes, coronary artery disease (CAD), heart failure, chronic obstructive pulmonary disease (COPD), asthma, cancer, obesity, and hyperlipidemia. In this context, each ribbon in the chord diagram indicates the normalized proportion of patients of a specific subphenotype with a specific comorbidity.

Supplementary Results – Determination of optimal cluster number

A. Subphenotype derivation in development cohort

To derive subphenotype in development cohort, dendrogram showed that the 4-cluster model is the optimal fit of the agglomerative hierarchical clustering model (see Supplementary Fig. 3). In addition, out of 21 indices in 'NbClust', 6 suggested 4 clusters, 5 suggested 2 clusters, 4 suggested 3 clusters, and 3 suggested 8 clusters. In conclusion, the optimal cluster number was 4.

B. Sensitivity analyses in development cohort

Sensitivity to quality control and outliers. A total of 140 patients with outlier values were excluded for re-deriving subphenotypes in development cohort. Performing agglomerative hierarchical clustering on the all 30 clinical variables of the remaining 8059 patients in development cohort, dendrogram showed that the 4-cluster model is the optimal fit (see Supplementary Fig. 3). In addition, out of 21 indices in 'NbClust', 8 suggested 2 clusters, 6 suggested 4 clusters, and 4 suggested 3 clusters. Comprehensively considering the dendrogram and 'NbClust' indices, the optimal cluster number was 4.

Sensitivity to clustering methods. GMM was performed on the 23 clinical variables of all 8199 patients in development cohort. In GMM smaller AIC and BIC indicate better cluster structure in the data, while higher cluster membership probability of each data point indicates more robust result. By comprehensively considering the decreasing rates of AIC, BIC, and median of membership probability, we chose the optimal cluster number as 4 (see Supplementary Table 5).

C. Validation in internal validation cohort

To re-derive subphenotype in internal validation cohort, dendrogram also showed that the 4-cluster model is the optimal fit of the agglomerative hierarchical clustering model (see Supplementary Fig. 7). In addition, out of 21 indices in 'NbClust', 7 suggested 2 clusters, 7 suggested 3 clusters, and 6 suggested 4 clusters. Comprehensively considering the dendrogram and 'NbClust' indices, the optimal cluster number was 4.

D. Derivation of SDoH strata in development cohort

In the development cohort, 7862 patients who had SDoH data were used to derive SDoH strata. Dendrogram showed that the 3-cluster model is the optimal fit of the agglomerative hierarchical clustering model (see Supplementary Fig. 13). In addition, out of 21 indices in 'NbClust', 9 suggested 3 clusters, 6 suggested 2 clusters, 3 suggested 4 clusters, and 3 suggested 8 clusters. Therefore, the optimal cluster number was 4.

References

1. Troyanskaya, O., *et al.* Missing value estimation methods for DNA microarrays. *Bioinformatics* **17**, 520-525 (2001).
2. Murtagh, F. & Legendre, P. Ward's Hierarchical Agglomerative Clustering Method: Which Algorithms Implement Ward's Criterion? *J. Classif.* **31**, 274-295 (2014).
3. Charrad, M., Ghazzali, N., Boiteau, V., Niknafs, A. & Charrad, M.M. Package 'nbclust'. *J. Stat. Softw.* **61**, 1-36 (2014).
4. Krzanowski, W.J. & Lai, Y.T. A Criterion for Determining the Number of Groups in a Data Set Using Sum-of-Squares Clustering. *Biometrics* **44**, 23-34 (1988).
5. Caliński, T. & Harabasz, J. A dendrite method for cluster analysis. *Communications in Statistics* **3**, 1-27 (1974).
6. Hartigan, J.A. *Clustering Algorithms*, (John Wiley & Sons, Inc., 1975).
7. Sarle, W.S. *Cubic clustering criterion*, (SAS institute, 1983).
8. Scott, A.J. & Symons, M.J. Clustering Methods Based on Likelihood Ratio Criteria. *Biometrics* **27**, 387-397 (1971).
9. Marriott, F.H.C. Practical Problems in a Method of Cluster Analysis. *Biometrics* **27**, 501-514 (1971).
10. Milligan, G.W. & Cooper, M.C. An examination of procedures for determining the number of clusters in a data set. *Psychometrika* **50**, 159-179 (1985).
11. Friedman, H.P. & Rubin, J. On Some Invariant Criteria for Grouping Data. *Journal of the American Statistical Association* **62**, 1159-1178 (1967).
12. Hubert, L.J. & Levin, J.R. A general statistical framework for assessing categorical clustering in free recall. *Psychol. Bull.* **83**, 1072 (1976).
13. Davies, D.L. & Bouldin, D.W. A Cluster Separation Measure. *IEEE Transactions on Pattern Analysis and Machine Intelligence* **PAMI-1**, 224-227 (1979).
14. Kaufman, L. & Rousseeuw, P.J. *Finding groups in data: an introduction to cluster analysis*, (John Wiley & Sons, 2009).
15. Duda, R.O. & Hart, P.E. *Pattern classification and scene analysis*, (Wiley New York, 1973).
16. Beale, E. *Euclidean cluster analysis*, (Scientific Control Systems Limited, 1969).
17. Ratkowsky, D. & Lance, G. Criterion for determining the number of groups in a classification. *Australian Computer Journal* **10**, 115-117 (1978).
18. Ball, G.H. & Hall, D.J. ISODATA, a novel method of data analysis and pattern classification. (Stanford research inst Menlo Park CA, 1965).
19. Milligan, G.W. An examination of the effect of six types of error perturbation on fifteen clustering algorithms. *Psychometrika* **45**, 325-342 (1980).
20. Frey, T. & van Groenewoud, H. A Cluster Analysis of the D2 Matrix of White Spruce Stands in Saskatchewan Based on the Maximum-Minimum Principle. *J. Ecol.* **60**, 873-886 (1972).
21. McClain, J.O. & Rao, V.R. CLUSTISZ: A Program to Test for the Quality of Clustering of a Set of Objects. *Journal of Marketing Research* **12**, 456-460 (1975).
22. Dunn†, J.C. Well-Separated Clusters and Optimal Fuzzy Partitions. *Journal of Cybernetics* **4**, 95-104 (1974).
23. Hubert, L. & Arabie, P. Comparing partitions. *J. Classif.* **2**, 193-218 (1985).
24. Halkidi, M., Vazirgiannis, M. & Batistakis, Y. Quality Scheme Assessment in the Clustering Process. in *Principles of Data Mining and Knowledge Discovery* (eds. Zighed, D.A., Komorowski, J. & Żytkow, J.) 265-276 (Springer Berlin Heidelberg, Berlin, Heidelberg, 2000).
25. Lebart, L., Morineau, A. & Piron, M. *Statistique exploratoire multidimensionnelle*, (Dunod Paris, 1995).

26. Halkidi, M. & Vazirgiannis, M. Clustering validity assessment: finding the optimal partitioning of a data set. in *Proceedings 2001 IEEE International Conference on Data Mining* 187-194 (2001).
27. Reynolds, D. Gaussian Mixture Models. in *Encyclopedia of Biometrics* (eds. Li, S.Z. & Jain, A.) 659-663 (Springer US, Boston, MA, 2009).
28. McInnes, L., Healy, J. & Melville, J. Umap: Uniform manifold approximation and projection for dimension reduction. *arXiv preprint arXiv:1802.03426* (2018).
29. Gu, Z., Gu, L., Eils, R., Schlesner, M. & Brors, B. circlize implements and enhances circular visualization in R. *Bioinformatics* **30**, 2811-2812 (2014).

Supplementary Fig. 1. Inclusion-exclusion criteria

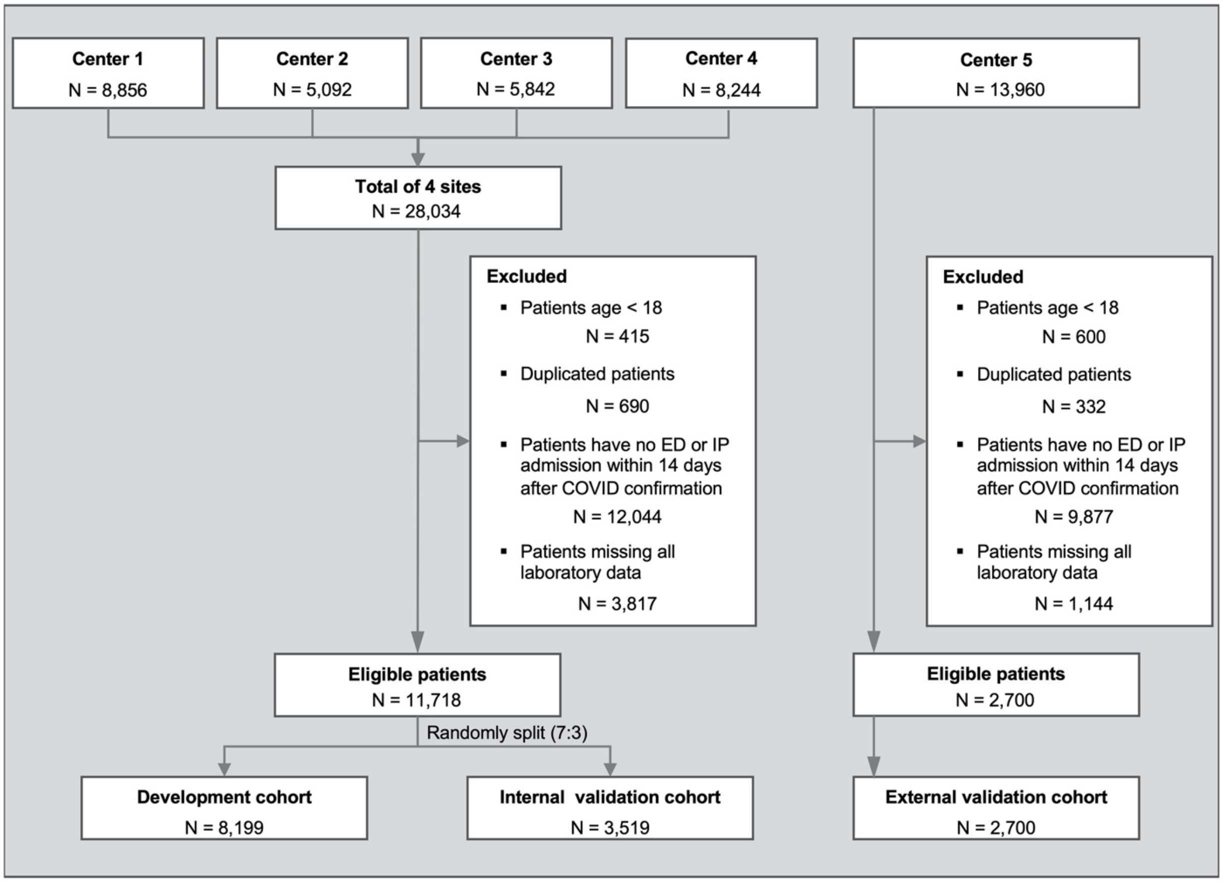

Supplementary Fig. 2. Method for extracting clinical data of patients

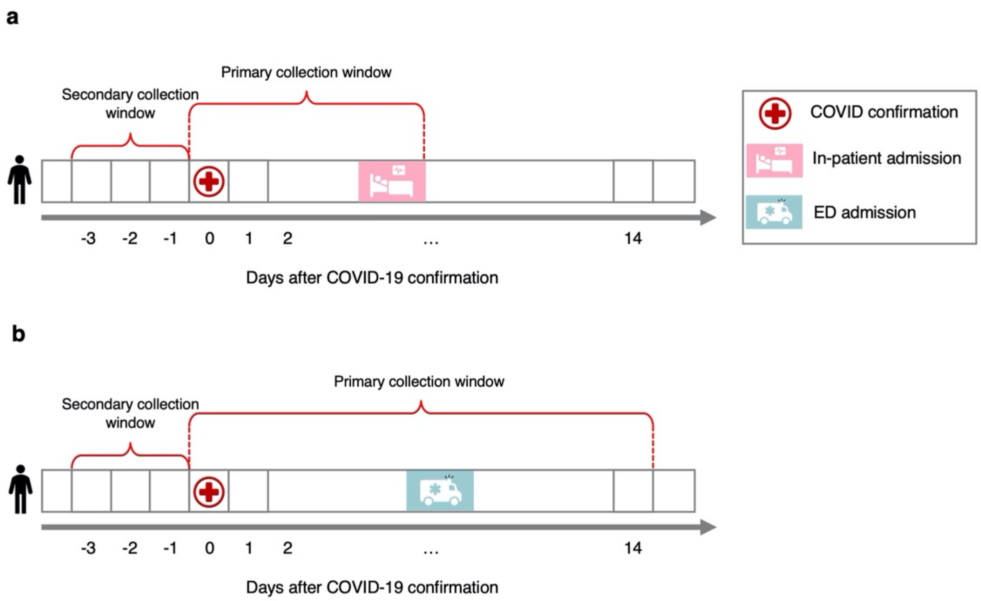

The primary collection window was defined as **(a)** time period from COVID-19 confirmation to the first in-patient encounter, if the patient has in-patient admission within 14 days after confirmation; or **(b)** 14 days after COVID-19 confirmation otherwise. We extracted the first value of each clinical variable within the primary collection window for each patient. If there was no record in the primary collection window, we extracted the last value within 3 days before confirmation (secondary collection window).

Abbreviations: ED = emergency department

Supplementary Fig. 3. Dendrogram for the subphenotypes in the development cohort

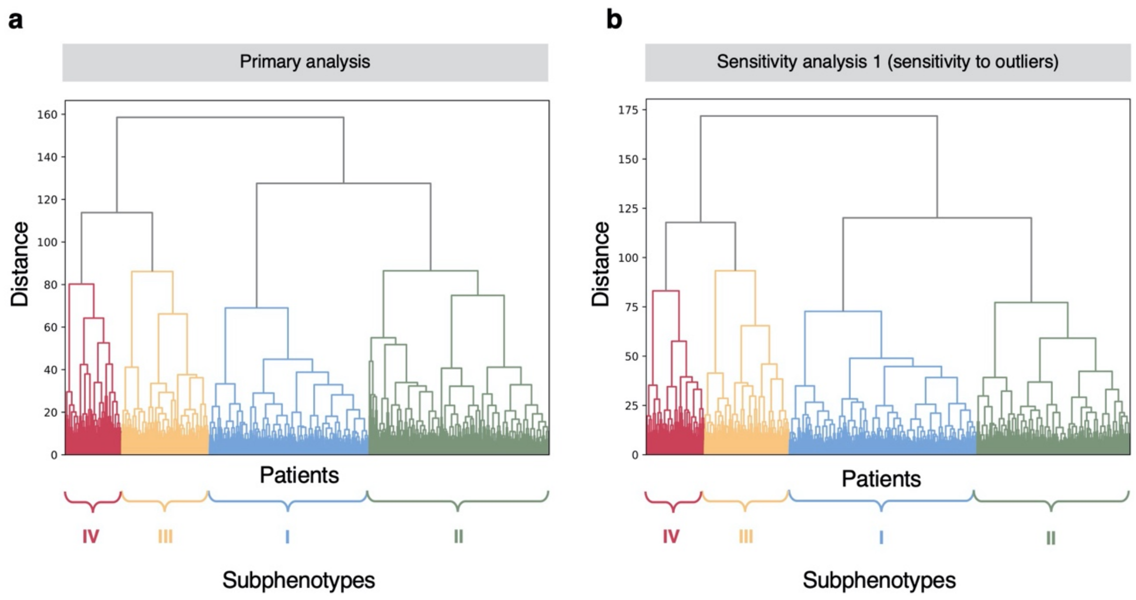

Supplementary Fig. 4. UMAP-based visualization of the subphenotypes in the development cohort

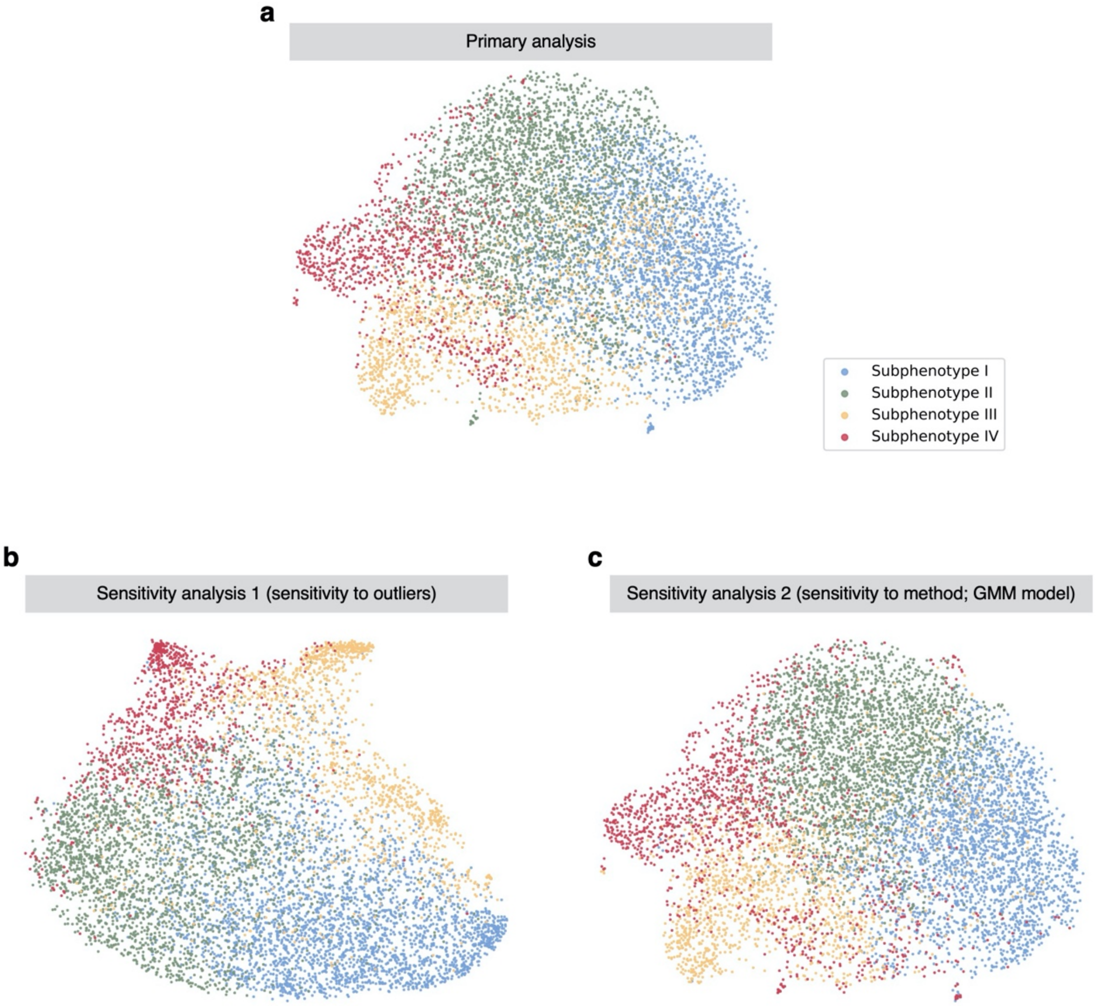

Abbreviations: UMAP = uniform manifold approximation and projection for dimension reduction.

Supplementary Fig. 5. ICU admission and intubation outcomes by subphenotype in the development cohort

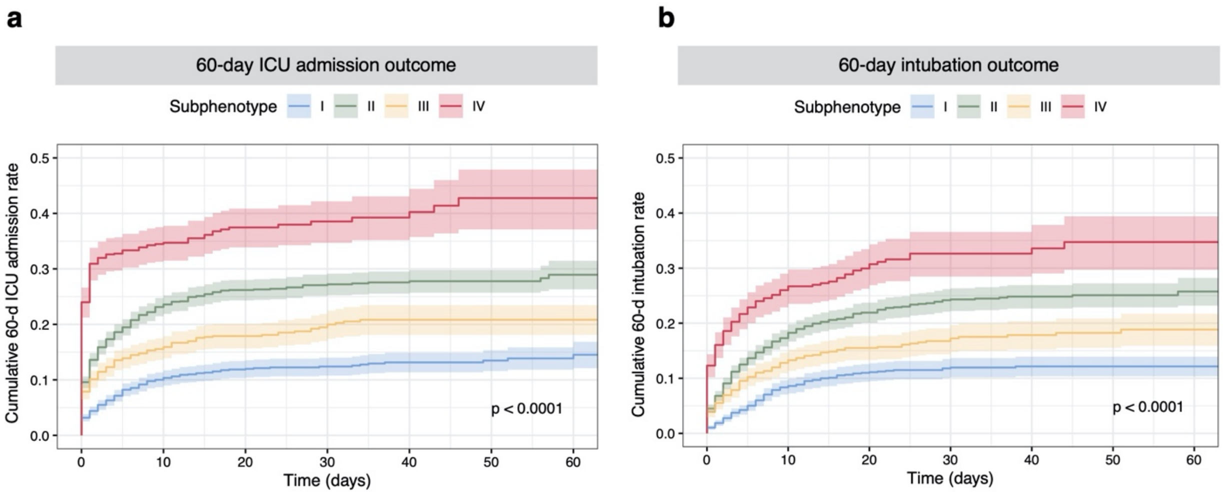

Supplementary Fig. 6. Confusion matrices for comparing the subphenotypes derived by primary analysis and sensitivity analyses in the development cohort

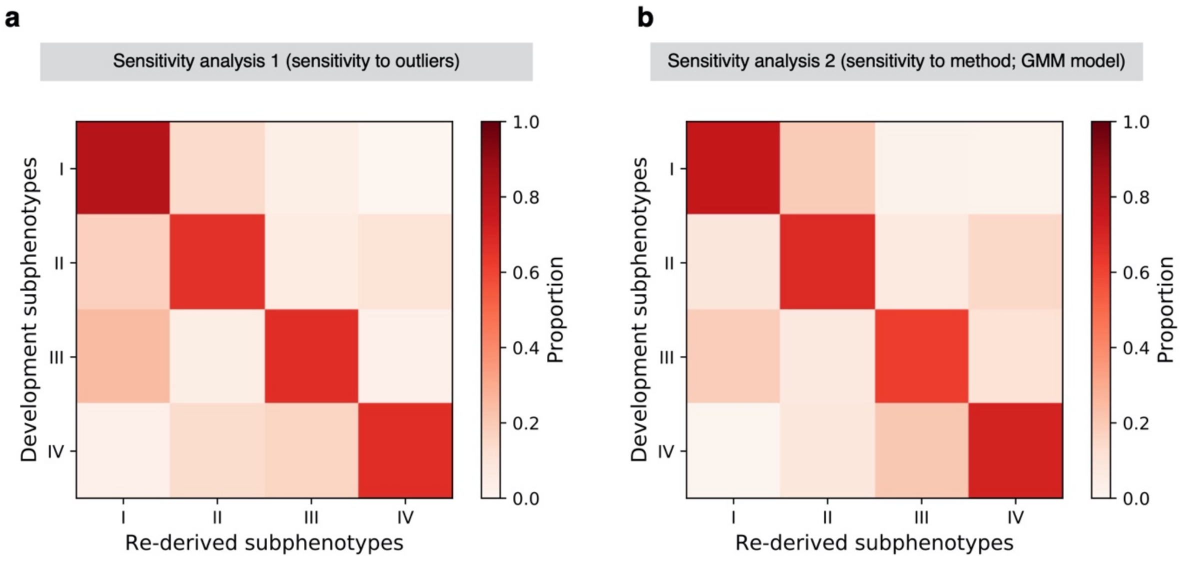

Supplementary Fig. 7. Dendrogram and UMAP-based visualization for the subphenotypes in the internal validation cohort

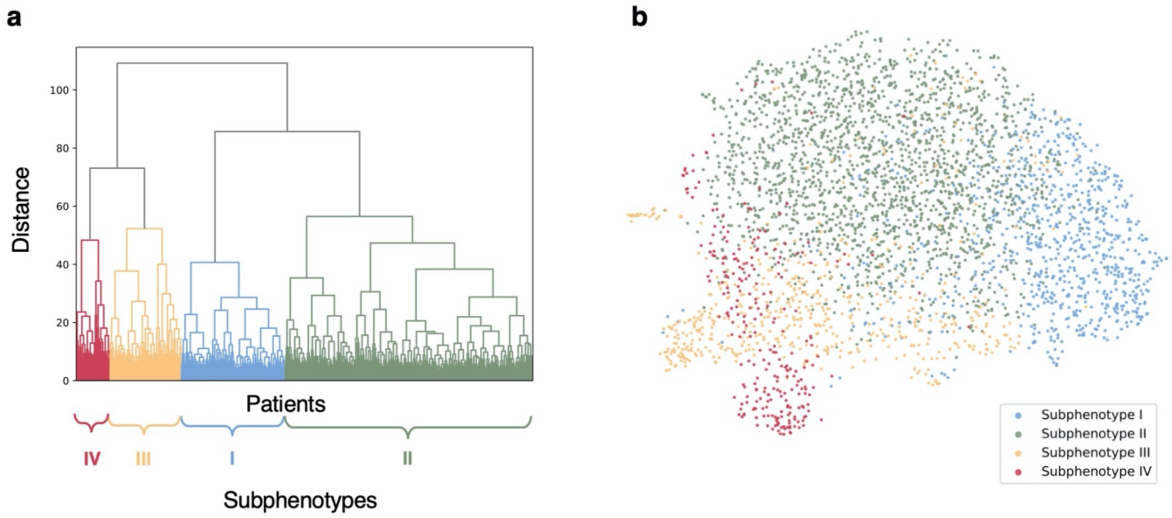

Abbreviations: UMAP = uniform manifold approximation and projection for dimension reduction.

Supplementary Fig. 8. Chord diagrams showing abnormal clinical variables and comorbidities by subphenotype in the internal validation cohort

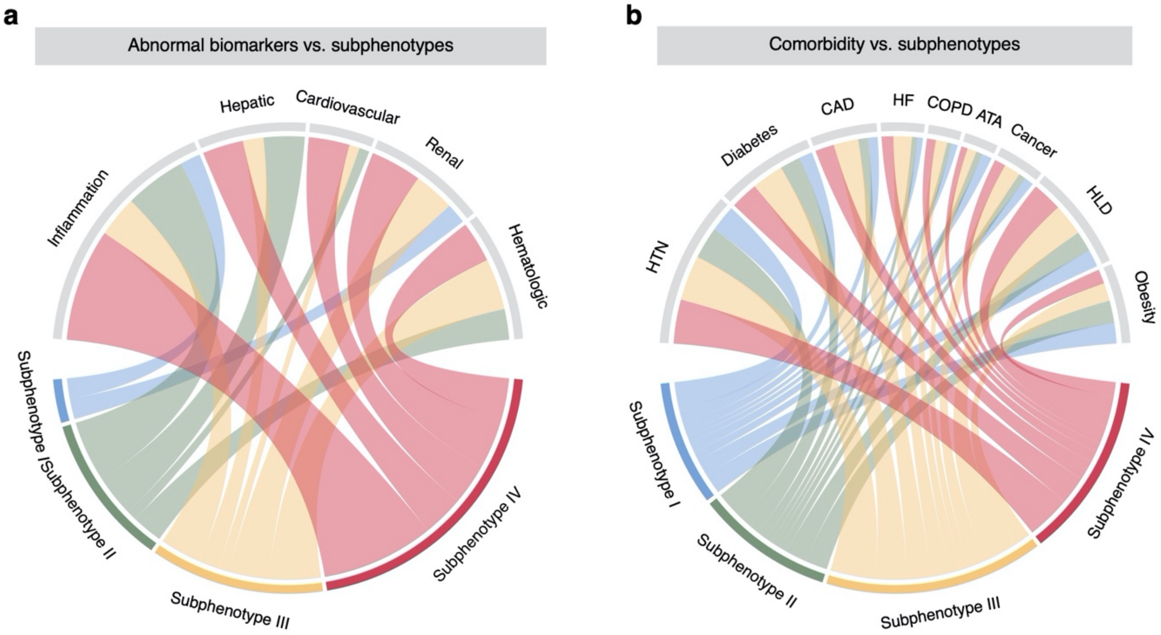

Abbreviations: ATA = asthma; CAD = coronary artery disease; COPD = chronic obstructive pulmonary disease; HF = heart failure; HLD = hyperlipidemia; HTN = hypertension.

Supplementary Fig. 9. Receiver operating characteristic (ROC) curves of the subphenotype prediction model in the development cohort

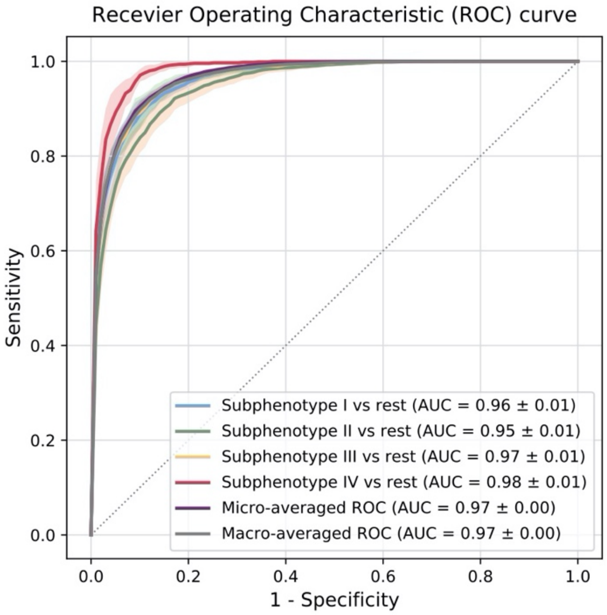

A macro-average typically computes the metric independently for each class and then takes the average (hence treating all classes equally), whereas a micro-average tries to aggregate the contributions of all classes to compute the average metric.

Abbreviations: AUC = area under the receiver operating characteristic curve.

Supplementary Fig. 10. SHAP value-based predictor contribution visualization of the subphenotype prediction model

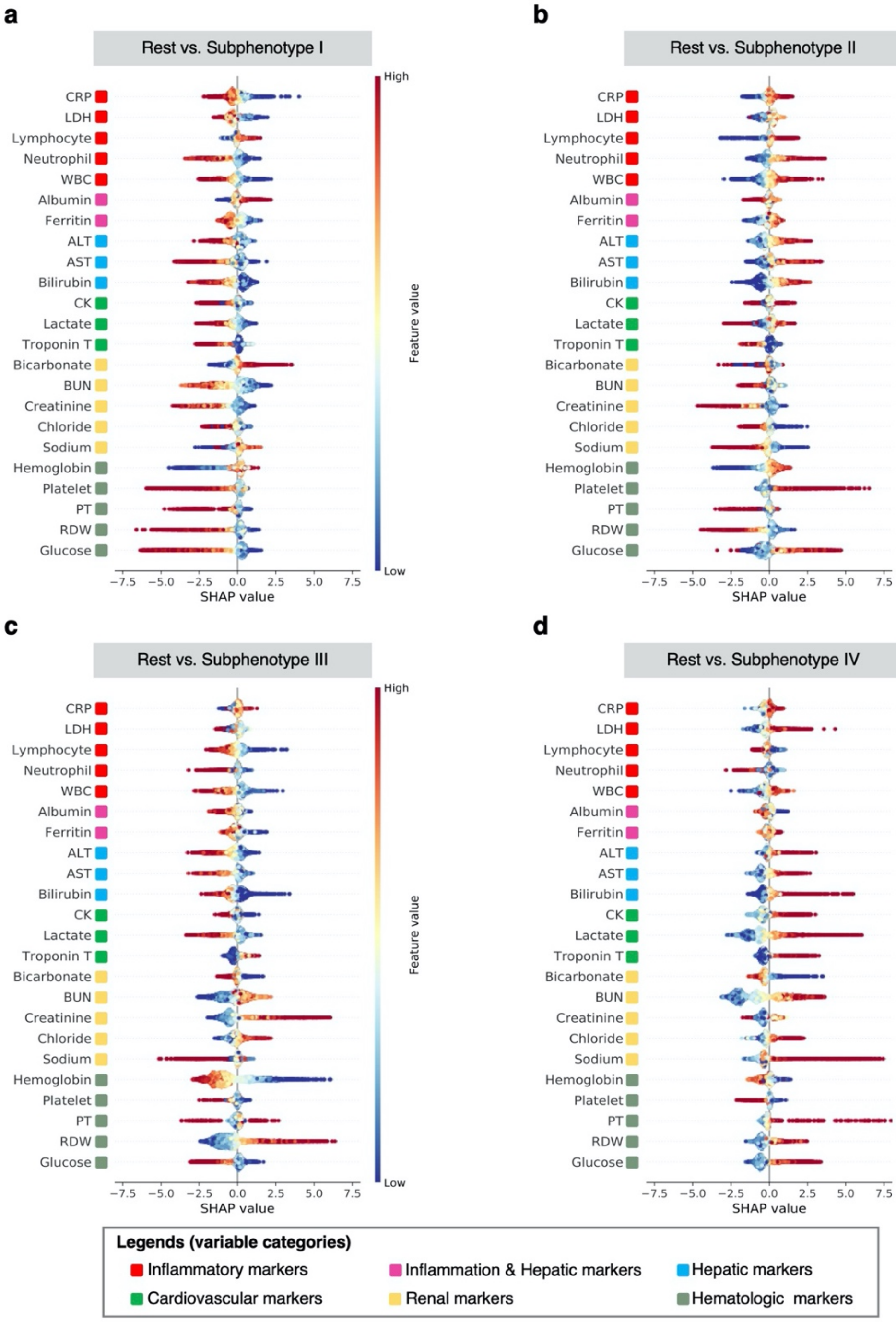

Abbreviations: ALT = alanine aminotransferase; AST = aspartate aminotransferase; BUN = blood urea nitrogen; CK = creatine kinase; CRP = C-reactive protein; ESR = Erythrocyte sedimentation rate; LDH = Lactate dehydrogenase; PT = prothrombin time; RDW = red blood cell distribution width; WBC = white blood cell count

Supplementary Fig. 11. UMAP-based visualization for the subphenotypes in the external validation cohort

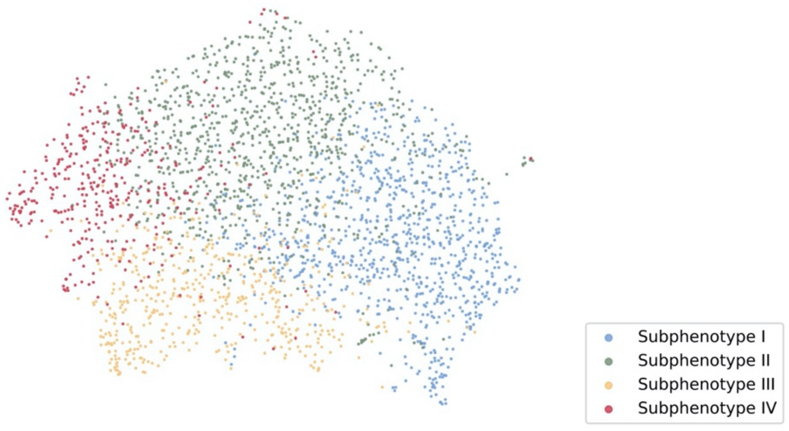

Abbreviations: UMAP = uniform manifold approximation and projection for dimension reduction.

Supplementary Fig. 12. Chord diagrams showing abnormal clinical variables and comorbidities by subphenotype in the external validation cohort

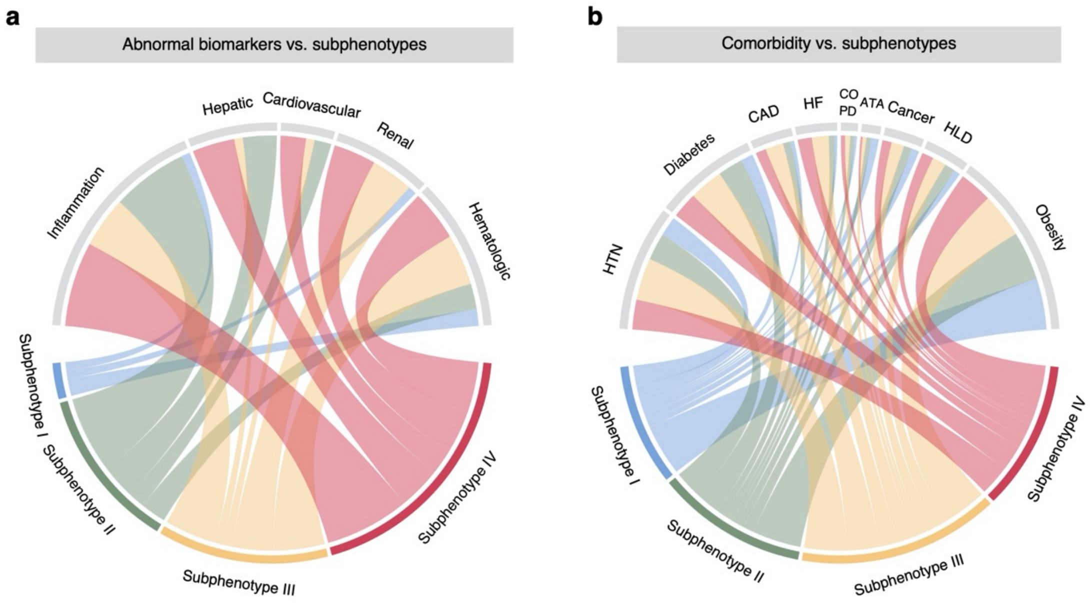

Abbreviations: ATA = asthma; CAD = coronary artery disease; COPD = chronic obstructive pulmonary disease; HF = heart failure; HLD = hyperlipidemia; HTN = hypertension.

Supplementary Fig. 13. Consensus matrix showing clusters obtained in leave-one-center-out cross-validation. Density of color denotes 'consensus value' between each pair of patients, calculated based on the frequency that these two patients fall into same cluster in the leave-one-center-out procedure. A consensus value of 0 means that the pair of patients never been grouped into the same cluster, while a value of 1 means that they always assigned to the same cluster during the leave-one-center-out procedure. By agglomerative hierarchical clustering, we identified clear four-cluster structure, demonstrating stability of the clusters (i.e., subphenotypes) across the five studied centers.

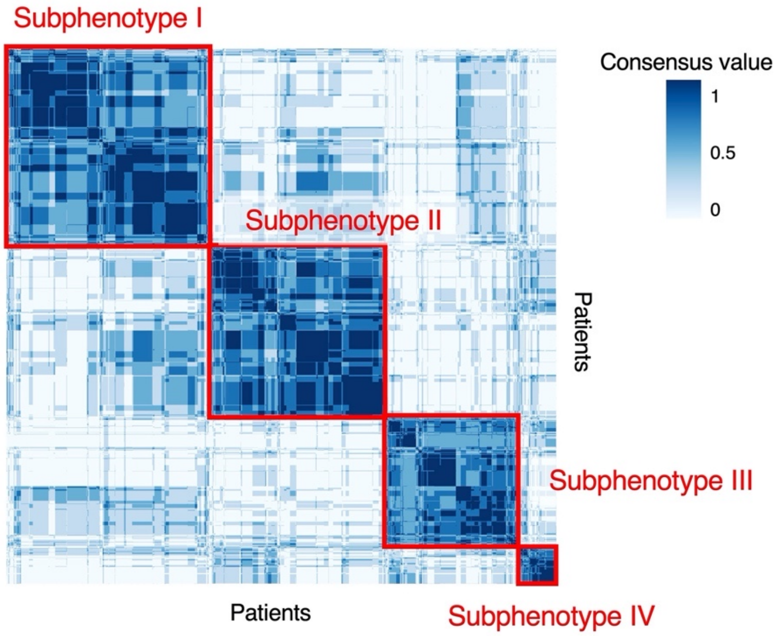

Supplementary Fig. 14. Dendrogram for SDoH strata identification in the development cohort

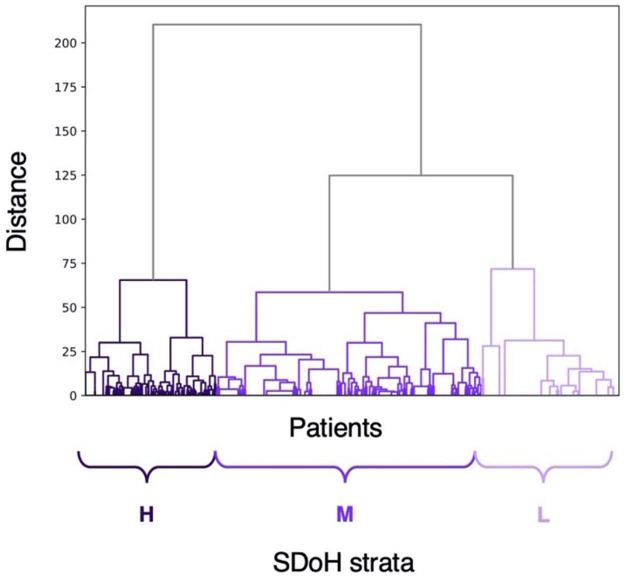

Abbreviations: SDoH = social determinants of health.

Supplementary Table 1. Characteristics of included patients in the five medical centers.

Variable	Medical centers				
	Center 1	Center 2	Center 3	Center 4	Center 5
No. of patients	4348	1215	314	5841	2700
**Demographics**					
Age, y, Median [IQR]	63.60 [49.96 - 75.18]	63.08 [49.15 - 76.23]	60.00 [45.55 - 71.20]	63.85 [51.59 - 75.13]	65.58 [51.08 - 77.39]
Sex female, N (%)	1878 (43.2%)	565 (46.5%)	157 (50.0%)	2772 (47.5%)	1305 (48.33)
Race, N (%)					
White	1874 (43.1%)	366 (30.1%)	65 (20.7%)	569 (9.7%)	675 (25.00)
Black	697 (16.0%)	169 (13.9%)	72 (22.9%)	2132 (36.5%)	545 (20.19)
Asian	279 (6.4%)	162 (13.3%)	9 (2.9%)	152 (2.6%)	28 (1.04)
Other/unknown	1498 (34.5%)	518 (42.6%)	168 (53.5%)	2988 (51.2%)	1452 (53.78)
**Patient care**					
Hospitalized patients[a], N (%)	641 (14.7%)	136 (11.2%)	198 (63.1%)	1016 (17.4%)	363 (13.4%)
Patients presented to ED without hospitalization[b], N (%)	3707 (85.3%)	1079 (88.8%)	116 (36.9%)	4825 (82.6%)	2337 (86.6%)

Abbreviations: ED = emergency department; IQR = Interquartile range.

[a] Patients who hospitalized within 14 days after COVID-19 confirmation.

[b] Patients who only had ED admission within 14 days after COVID-19 confirmation.

Supplementary Table 2. Presenting value distribution of candidate clinical variables for cluster analysis

Variable	Development cohort	Internal validation cohort	External validation cohort
Albumin, g/dL, Median [IQR]	3.70 [3.30 - 4.10]	3.70 [3.30 - 4.10]	3.80 [3.40 - 4.10]
Alanine aminotransferase, U/L, Median [IQR]	29.00 [19.00 - 48.00]	30.00 [19.00 - 49.00]	39.00 [26.00 - 65.00]
Aspartate aminotransferase, U/L, Median [IQR]	39.00 [26.00 - 63.00]	40.00 [27.00 - 64.00]	27.00 [17.00 - 47.00]
Bands, %, Median [IQR]	2.00 [0.00 - 5.00]	2.00 [1.00 - 6.00]	3.00 [2.00 - 5.00]
Bicarbonate, mmol/L, Median [IQR]	23.00 [21.00 - 26.00]	23.00 [21.00 - 26.00]	23.00 [20.00 - 25.00]
Bilirubin, mg/dL, Median [IQR]	0.30 [0.20 - 0.60]	0.30 [0.20 - 0.60]	0.30 [0.20 - 0.50]
BUN, mg/dL, Median [IQR]	17.00 [11.00 - 31.00]	17.00 [11.00 - 29.00]	18.00 [12.00 - 33.00]
Creatine kinase, U/L, Median [IQR]	154.00 [78.00 - 359.00]	151.00 [80.50 - 335.50]	154.50 [79.00 - 347.75]
Creatinine, mg/dL, Median [IQR]	1.00 [0.80 - 1.50]	1.00 [0.80 - 1.50]	1.10 [0.80 - 1.60]
Chloride, mmol/L, Median [IQR]	100.00 [97.00 - 104.00]	100.00 [97.00 - 104.00]	100.00 [96.00 - 103.00]
C-reactive protein, mg/L, Median [IQR]	9.40 [3.70 – 16.80]	9.63 [4.39 – 16.42]	11.30 [5.15 – 20.15]
D-dimer, ng/mL, Median [IQR]	1360.00 [620.00 - 3370.00]	1330.00 [585.25 - 3092.50]	1400.00 [700.00 - 3100.00]
ESR, mm/hr, Median [IQR]	69.00 [42.00 - 97.00]	64.00 [41.00 - 93.00]	72.00 [50.00 - 97.00]
Ferritin, ng/mL, Median [IQR]	645.00 [295.90 - 1347.00]	699.50 [297.00 - 1435.00]	672.65 [333.03 - 1276.00]
Glucose, mg/dL, Median [IQR]	121.00 [101.00 - 165.00]	122.00 [101.50 - 164.00]	125.00 [104.00 - 176.00]
Hemoglobin, g/dL, Median [IQR]	13.10 [11.50 - 14.60]	13.30 [11.70 - 14.70]	13.00 [11.40 - 14.30]
IL-6, pg/mL, Median [IQR]	19.00 [10.00 - 42.00]	16.50 [9.00 - 35.25]	45.20 [11.15 - 108.40]
Lactate, mmol/L, Median [IQR]	1.90 [1.40 - 2.60]	1.90 [1.30 - 2.60]	1.60 [1.20 - 2.30]
LDH, U/L, Median [IQR]	377.00 [280.00 - 525.00]	382.00 [285.00 - 527.00]	419.00 [310.50 - 599.50]
Lymphocyte count, $\times 10^3$/uL, Median [IQR]	1.00 [0.70 - 1.43]	1.00 [0.70 - 1.40]	1.00 [0.70 - 1.50]
Neutrophil count, $\times 10^3$/uL, Median [IQR]	5.30 [3.70 - 7.90]	5.30 [3.70 - 7.70]	5.40 [3.70 - 8.10]
Oxygen saturation, %, Median [IQR]	69.00 [50.00 - 85.00]	72.00 [48.50 - 90.00]	97.10 [92.00 - 98.70]
Prothrombin time, s, Median [IQR]	13.30 [12.20 - 14.60]	13.20 [12.20 - 14.40]	14.20 [13.40 - 15.30]
Procalcitonin, ng/mL, Median [IQR]	0.20 [0.10 - 0.60]	0.20 [0.10 - 0.50]	0.20 [0.10 - 0.60]
Platelet count, $\times 10^3$/uL, Median [IQR]	211.00 [162.00 - 277.00]	210.00 [163.00 - 274.00]	203.00 [157.00 - 262.50]
Red blood cell distribution width, %, Median [IQR]	13.80 [12.90 - 15.00]	13.70 [13.00 - 14.90]	13.90 [13.00 - 15.00]
Sodium, mmol/L, Median [IQR]	137.00 [134.00 - 140.00]	137.00 [134.00 - 140.00]	138.00 [135.00 - 141.00]
Troponin I, ng/mL, Median [IQR]	0.10 [0.06 - 0.30]	0.10 [0.07 - 0.30]	0.00 [0.00 - 0.10]

Troponin T, ng/mL, Median [IQR]	0.01 [0.01 - 0.03]	0.01 [0.01 - 0.02]	0.02 [0.01 - 0.04]
White blood cell count, $\times 10^3$/uL, Median [IQR]	7.20 [5.30 - 9.90]	7.10 [5.30 - 9.60]	7.30 [5.40 - 10.20]

Abbreviations: BUN = blood urea nitrogen; ESR = Erythrocyte sedimentation rate; IL-6 = Interleukin 6; IQR = Interquartile range; LDH = Lactate dehydrogenase.

Supplementary Table 3. Missing values (n (%)) across cohorts for candidate clinical variables at presentation

Variable	Development cohort	Internal validation cohort	External validation cohort
No. of patients	8,199	3,519	2,700
Albumin	915 (11.2%)	404 (11.5%)	476 (17.6%)
Alanine aminotransferase	961 (11.7%)	422 (12.0%)	474 (17.6%)
Aspartate aminotransferase	1383 (16.9%)	613 (17.4%)	467 (17.3%)
Bands	6435 (78.5%)	2805 (79.7%)	2467 (91.4%)
Bicarbonate	254 (3.1%)	138 (3.9%)	188 (7.0%)
Bilirubin	989 (12.1%)	442 (12.6%)	474 (17.6%)
BUN	209 (2.5%)	115 (3.3%)	195 (7.2%)
Creatine kinase	3958 (48.3%)	1752 (49.8%)	1394 (51.6%)
Creatinine	202 (2.5%)	111 (3.2%)	187 (6.9%)
Chloride	364 (4.4%)	195 (5.5%)	187 (6.9%)
C-reactive protein	3697 (45.1%)	1617 (46.0%)	1109 (41.1%)
D-dimer	6119 (74.6%)	2651 (75.3%)	1869 (69.2%)
ESR	6834 (83.4%)	2907 (82.6%)	1465 (54.3%)
Ferritin	4120 (50.3%)	1814 (51.5%)	1240 (45.9%)
Glucose	199 (2.4%)	92 (2.6%)	143 (5.3%)
Hemoglobin	52 (0.6%)	28 (0.8%)	103 (3.8%)
IL-6	7685 (93.7%)	3287 (93.4%)	2380 (88.1%)
Lactate	3444 (42.0%)	1498 (42.6%)	1129 (41.8%)
LDH	5422 (66.1%)	2326 (66.1%)	1197 (44.3%)
Lymphocyte count	659 (8.0%)	277 (7.9%)	275 (10.2%)
Neutrophil count	679 (8.3%)	287 (8.2%)	272 (10.1%)
Oxygen saturation	7604 (92.7%)	3236 (92.0%)	2465 (91.3%)
Prothrombin time	5552 (67.7%)	2397 (68.1%)	1105 (40.9%)
Procalcitonin	5973 (72.9%)	2568 (73.0%)	1035 (38.3%)
Platelet count	88 (1.1%)	47 (1.3%)	129 (4.8%)
Red blood cell distribution width	82 (1.0%)	41 (1.2%)	119 (4.4%)
Sodium	198 (2.4%)	107 (3.0%)	176 (6.5%)
Troponin T	4939 (60.2%)	2157 (61.3%)	831 (30.8%)
Troponin I	7236 (88.3%)	3114 (88.5%)	2597 (96.2%)
White blood cell count	78 (1.0%)	40 (1.1%)	119 (4.4%)

Abbreviations: BUN = blood urea nitrogen; ESR = Erythrocyte sedimentation rate; IL-6 = Interleukin 6; IQR = Interquartile range; LDH = Lactate dehydrogenase.

Supplementary Table 4. Usage of the candidate clinical variables in analysis

Variable	**Usage of the clinical variable in analysis**<br>D: Used for subphenotype derivation (development cohort).<br>S: Used for sensitivity analysis.<br>R: Used for re-derivation of subphenotypes (validation cohort).<br>P: Used for building prediction model of subphenotypes	Logarithmic transform?
Albumin	D, S, R, P	
Alanine aminotransferase	D, S, R, P	yes
Aspartate aminotransferase	D, S, R, P	yes
Bands	S	yes
Bicarbonate	D, S, R, P	
Bilirubin	D, S, R, P	yes
BUN	D, S, R, P	yes
Creatine kinase	D, S, R, P	yes
Creatinine	D, S, R, P	yes
Chloride	D, S, R, P	yes
C-reactive protein	D, S, R, P	yes
D-dimer	S	yes
ESR	S	
Ferritin	D, S, R, P	yes
Glucose	D, S, R, P	yes
Hemoglobin	D, S, R, P	yes
IL-6	S	yes
Lactate	D, S, R, P	yes
LDH	D, S, R, P	yes
Lymphocyte count	D, S, R, P	yes
Neutrophil count	D, S, R, P	
Oxygen saturation	S	
Prothrombin time	D, S, R, P	yes
Procalcitonin	S	yes
Platelet count	D, S, R, P	
Red blood cell distribution width	D, S, R, P	
Sodium	D, S, R, P	yes
Troponin T	D, S, R, P	yes
Troponin I	S	yes
White blood cell count	D, S, R, P	yes

Abbreviations: BUN = blood urea nitrogen; ESR = Erythrocyte sedimentation rate; IL-6 = Interleukin 6; IQR = Interquartile range; LDH = Lactate dehydrogenase.

Supplementary Table 5. Statistical output from Gaussian mixture model for sensitivity analysis 2 in development cohort

Cluster number	BIC	AIC	Membership probability, median [IQR]
2	469836.062	469492.486	99.99 [99.44 - 100.00]
3	459066.27	458547.399	99.74 [94.16 - 100.00]
4	454755.227	454061.062	99.11 [88.76 - 99.97]
5	449328.294	448458.834	98.22 [85.53 - 99.90]
6	446186.472	445141.719	97.29 [82.43 - 99.83]
7	443065.314	441845.266	97.43 [82.84 - 99.84]
8	440346.512	438951.17	97.30 [82.37 - 99.81]

Abbreviations: IQR = Interquartile range

Supplementary Table 6. Characteristics of the subphenotypes in sensitivity analysis 1 (quality control and outliers) in the development cohort.

Variable	Total	Subphenotype I	Subphenotype II	Subphenotype III	Subphenotype IV	P-value[a]	P-value (age and sex adjusted)[b]
No. of patients (%)	8059 (100)[c]	3125 (38.78)	2549 (31.63)	1413 (17.53)	972 (12.06)	-	-
**Demographics**							
Age, y, Median (IQR)	63.53 (50.54 - 75.17)	60.47 (46.11 - 73.36)	61.10 (49.95 - 71.13)	67.63 (55.55 - 79.35)	72.92 (62.46 - 82.62)	< 0.001	-
Sex female, N (%)	3723 (46.20)	1723 (55.14)	908 (35.62)	695 (49.19)	397 (40.84)	< 0.001	-
Race, N (%)							
White	2001 (24.83)	856 (27.39)	603 (23.66)	331 (23.43)	211 (21.71)	< 0.001	-
Black	2111 (26.19)	806 (25.79)	520 (20.40)	472 (33.40)	313 (33.20)		
Asian	399 (4.95)	138 (4.42)	174 (6.83)	53 (3.75)	34 (3.5)		
Other/unknown	3548 (44.02)	1325 (42.40)	1252 (49.12)	557 (39.42)	414 (42.60)		
**Inflammatory markers**							
C-reactive protein, mg/L, Median (IQR)	9.35 (3.78 - 16.77)	4.60 (1.40 - 9.35)	13.20 (7.44 - 19.56)	8.60 (3.20 - 15.49)	16.24 (7.57 - 25.10)	< 0.001	< 0.001
ESR, mm/hr, Median (IQR)	69.00 (42.00 - 97.00)	51.00 (33.00 - 76.00)	76.00 (51.00 - 99.00)	76.00 (47.00 - 106.25)	86.00 (50.50 - 108.00)	< 0.001	< 0.001
IL-6, pg/mL, Median (IQR)	18.00 (10.00 - 42.00)	13.00 (8.00 - 26.25)	17.50 (10.00 - 41.00)	20.00 (11.00 - 47.00)	37.00 (17.00 - 73.50)	< 0.001	< 0.001
Procalcitonin, ng/mL, Median (IQR)	0.20 (0.10 - 0.51)	0.10 (0.10 - 0.20)	0.20 (0.10 - 0.50)	0.40 (0.14 - 1.12)	0.64 (0.30 - 2.08)	< 0.001	< 0.001
Bands, %, Median (IQR)	2.00 (0.00 - 5.00)	3.00 (0.00 - 6.00)	2.00 (1.00 - 5.00)	2.00 (0.00 - 5.00)	2.00 (0.00 - 6.00)	0.32	0.09
LDH, U/L, Median (IQR)	376.00 (279.00 - 521.50)	301.00 (237.00 - 388.50)	433.00 (345.00 - 574.00)	341.00 (256.00 - 477.00)	567.00 (420.00 - 789.00)	< 0.001	< 0.001
Lymphocyte count, $\times 10^3$/uL, Median (IQR)	1.00 (0.70 - 1.40)	1.10 (0.80 - 1.60)	0.90 (0.60 - 1.30)	0.90 (0.60 - 1.30)	1.00 (0.70 - 1.50)	< 0.001	< 0.001
Neutrophil count, $\times 10^3$/uL, Median (IQR)	5.30 (3.60 - 7.80)	4.20 (3.00 - 5.60)	6.10 (4.30 - 8.50)	5.30 (3.70 - 7.40)	10.41 (7.50 - 13.78)	< 0.001	< 0.001
White blood cell count, $\times 10^3$/uL, Median (IQR)	7.20 (5.30 - 9.80)	6.00 (4.70 - 7.70)	7.90 (6.00 - 10.30)	7.00 (5.20 - 9.50)	12.70 (9.50 - 16.50)	< 0.001	< 0.001
**Inflammation & Hepatic markers**							
Albumin, g/dL, Median (IQR)	3.70 (3.30 - 4.10)	3.90 (3.60 - 4.30)	3.70 (3.30 - 4.00)	3.40 (2.90 - 3.90)	3.40 (2.90 - 3.73)	< 0.001	< 0.001
Ferritin, ng/mL, Median (IQR)	643.00 (296.00 - 1339.60)	351.55 (173.97 - 642.35)	958.00 (503.80 - 1666.70)	621.20 (212.00 - 1506.10)	1026.10 (519.00 - 1987.25)	< 0.001	< 0.001
**Hepatic markers**							
Alanine aminotransferase, U/L, Median (IQR)	29.00 (19.00 - 48.00)	24.00 (17.00 - 34.00)	45.00 (28.00 - 73.00)	20.00 (14.00 - 32.00)	39.00 (23.00 - 67.00)	< 0.001	< 0.001
Aspartate aminotransferase, U/L, Median (IQR)	39.00 (26.00 - 63.00)	31.00 (23.00 - 41.00)	57.00 (39.00 - 84.00)	32.00 (21.00 - 50.00)	62.00 (36.00 - 113.00)	< 0.001	< 0.001
Bilirubin, mg/dL, Median (IQR)	0.30 (0.20 - 0.60)	0.30 (0.20 - 0.50)	0.40 (0.20 - 0.60)	0.30 (0.20 - 0.50)	0.40 (0.20 - 0.60)	< 0.001	< 0.001
**Cardiovascular markers**							
Creatine kinase, U/L, Median (IQR)	153.00 (78.00 - 353.00)	123.00 (75.00 - 217.25)	190.00 (96.00 - 441.50)	117.00 (57.50 - 283.50)	305.00 (110.00 - 1041.00)	< 0.001	< 0.001
Lactate, mmol/L, Median (IQR)	1.80 (1.38 - 2.60)	1.60 (1.20 - 2.12)	1.90 (1.50 - 2.50)	1.70 (1.30 - 2.30)	3.10 (2.20 - 4.70)	< 0.001	< 0.001
Troponin I, ng/mL, Median (IQR)	0.10 (0.06 - 0.30)	0.10 (0.01 - 0.10)	0.10 (0.04 - 0.28)	0.10 (0.10 - 0.30)	0.20 (0.10 - 0.50)	< 0.001	0.02
Troponin T, ng/mL, Median (IQR)	0.01 (0.01 - 0.03)	0.01 (0.01 - 0.01)	0.01 (0.01 - 0.01)	0.04 (0.01 - 0.12)	0.04 (0.01 - 0.12)	< 0.001	< 0.001
**Renal markers**							
Bicarbonate, mmol/L, Median (IQR)	23.00 (21.00 - 26.00)	24.00 (22.00 - 26.00)	23.00 (21.00 - 26.00)	23.00 (19.00 - 25.10)	20.00 (17.00 - 23.00)	< 0.001	< 0.001

BUN, mg/dL, Median (IQR)	17.00 (11.00 - 30.00)	14.00 (10.00 - 19.00)	15.00 (11.00 - 22.00)	30.00 (16.00 - 56.00)	47.00 (28.00 - 79.00)	< 0.001	< 0.001
Creatinine, mg/dL, Median (IQR)	1.00 (0.80 - 1.50)	0.90 (0.74 - 1.12)	0.97 (0.80 - 1.20)	1.70 (0.90 - 4.80)	1.85 (1.20 - 3.08)	< 0.001	< 0.001
Chloride, mmol/L, Median (IQR)	100.00 (97.00 - 104.00)	101.00 (98.00 - 104.00)	98.00 (95.00 - 101.00)	101.00 (96.00 - 105.00)	104.00 (98.00 - 112.00)	< 0.001	< 0.001
Sodium, mmol/L, Median (IQR)	137.00 (134.00 - 140.00)	138.00 (136.00 - 141.00)	135.00 (132.00 - 138.00)	137.00 (134.00 - 140.00)	141.00 (135.00 - 152.00)	< 0.001	< 0.001
**Hematologic markers**							
D-dimer, ng/mL, Median (IQR)	1345.00 (611.50 - 3332.50)	706.00 (380.00 - 1470.00)	1080.00 (590.00 - 2185.00)	2030.00 (990.00 - 3680.00)	4640.00 (2250.00 - 13960.00)	< 0.001	< 0.001
Hemoglobin, g/dL, Median (IQR)	13.20 (11.60 - 14.60)	13.40 (12.20 - 14.70)	13.80 (12.60 - 15.10)	10.60 (8.70 - 12.10)	13.20 (11.45 - 15.20)	< 0.001	< 0.001
Platelet count, $\times 10^3$/uL, Median (IQR)	211.00 (163.00 - 276.00)	201.00 (161.00 - 253.00)	212.00 (168.00 - 271.00)	211.00 (148.00 - 309.00)	253.00 (177.00 - 344.00)	< 0.001	< 0.001
Prothrombin time, s, Median (IQR)	13.20 (12.20 - 14.50)	12.80 (12.00 - 13.80)	13.40 (12.40 - 14.50)	13.30 (12.00 - 15.60)	14.40 (13.00 - 16.95)	< 0.001	< 0.001
Red blood cell distribution width, %, Median (IQR)	13.80 (12.90 - 15.00)	13.50 (12.80 - 14.40)	13.20 (12.60 - 14.10)	15.80 (14.20 - 17.90)	14.70 (13.70 - 16.10)	< 0.001	< 0.001
Glucose, mg/dL, Median (IQR)	121.00 (101.00 - 165.00)	110.00 (96.00 - 131.00)	134.00 (110.00 - 210.00)	117.00 (96.00 - 154.00)	158.00 (120.00 - 239.00)	< 0.001	< 0.001
**Other markers**							
Oxygen saturation, %, Median (IQR)	69.00 (50.00 - 85.00)	67.00 (49.00 - 85.00)	69.00 (52.00 - 85.00)	70.50 (48.00 - 80.75)	76.00 (53.25 - 91.65)	0.31	0.41
BMI, kg/m^2, Median (IQR)	28.00 (25.00 - 33.00)	29.00 (25.00 - 34.00)	29.00 (25.00 - 33.00)	27.00 (23.00 - 32.00)	26.00 (23.00 - 31.00)	< 0.001	0.62
**Comorbidity, (missing=579), N (%)**							
Hypertension	4664 (62.35)	1571 (54.34)	1373 (58.25)	1015 (76.89)	705 (77.30)	< 0.001	-
Diabetes	3057 (40.87)	867 (29.99)	1018 (43.19)	682 (51.67)	490 (53.73)	< 0.001	-
Coronary artery disease	1719 (22.98)	507 (17.54)	420 (17.82)	490 (37.12)	302 (33.11)	< 0.001	-
Heart failure	1099 (14.69)	290 (10.03)	197 (8.36)	418 (31.67)	194 (21.27)	< 0.001	-
COPD	945 (12.63)	345 (11.93)	190 (8.06)	259 (19.62)	151 (16.56)	< 0.001	-
Asthma	1069 (14.29)	441 (15.25)	318 (13.49)	217 (16.44)	93 (10.20)	< 0.001	-
Cancer	1388 (18.56)	497 (17.19)	307 (13.03)	396 (30.00)	188 (20.61)	< 0.001	-
Hyperlipidemia	3199 (42.77)	1078 (37.29)	947 (40.18)	725 (54.92)	449 (49.23)	< 0.001	-
Obesity	3006 (37.30)	1218 (38.98)	1018 (39.94)	484 (34.25)	286 (29.42)	< 0.001	-
**Outcomes (60 days), N (%)**							
Mortality	1472 (18.27)	266 (8.51)	411 (16.12)	340 (24.06)	455 (46.81)	< 0.001	-
Mechanical ventilation (intubation)	1115 (13.84)	222 (7.10)	436 (17.10)	197 (13.94)	260 (26.75)	< 0.001	-
ICU admission	1446 (17.94)	291 (9.31)	549 (21.54)	251 (17.76)	355 (36.52)	< 0.001	-
**Medications, N (%)**							
Antibiotics	2896 (35.93)	901 (28.83)	975 (38.25)	526 (37.23)	494 (50.82)	< 0.001	-
Corticosteroids	1628 (20.20)	415 (13.28)	595 (23.34)	294 (20.81)	324 (33.33)	< 0.001	-
Enoxaparin	3266 (40.53)	1203 (38.50)	1335 (52.37)	381 (26.96)	347 (35.70)	< 0.001	-
Heparin	1280 (15.88)	357 (11.42)	472 (18.52)	283 (20.03)	168 (17.28)	< 0.001	-
Vasopressor	582 (7.22)	138 (4.42)	248 (9.73)	100 (7.08)	96 (9.88)	< 0.001	-

Abbreviations: BUN = blood urea nitrogen; COPD = chronic obstructive pulmonary disease; ESR = Erythrocyte sedimentation rate; ICU = intensive care unit; IL-6 = Interleukin 6; IQR = Interquartile range; LDH = Lactate dehydrogenase.

[a] Comparisons across all 4 subphenotypes were performed using the Kruskal-Wallis test (with Dunn's test for post-hoc pairwise comparisons) or $\chi 2$ test.

[b] P-values, adjusting for age and sex, were calculated by analysis of covariance (ANCOVA) was performed based on General Linear Model.

[c] 140 patients in development cohort were excluded in the first sensitivity analysis as they had outlier data.

Supplementary Table 7. Characteristics of the subphenotypes in sensitivity analysis 2 (Gaussian mixture model) in the development cohort.

Variable	Total	Subphenotype I	Subphenotype II	Subphenotype III	Subphenotype IV	P-value[a]	P-value (age and sex adjusted)[b]
No. of patients (%)	8199 (100)	2641 (32.21)	2807 (34.24)	1395 (17.01)	1356 (16.54)	-	-
**Demographics**							
Age, y, Median (IQR)	63.53 (50.57 - 75.15)	57.05 (42.98 - 70.85)	62.17 (50.87 - 72.40)	71.50 (60.21 - 81.02)	68.59 (57.21 - 79.89)	< 0.001	-
Sex female, N (%)	3787 (46.19)	1614 (61.11)	981 (34.95)	618 (44.30)	574 (42.33)	< 0.001	-
Race, N (%)							
White	2036 (24.83)	684 (25.90)	741 (26.40)	325 (23.30)	286 (21.09)	< 0.001	-
Black	2155 (26.28)	720 (27.26)	521 (18.56)	488 (34.98)	426 (31.42)		
Asian	409 (4.99)	96 (3.63)	189 (6.73)	60 (4.30)	64 (4.72)		
Other/unknown	3599 (43.90)	1141 (43.20)	1356 (48.31)	522 (37.42)	580 (42.77)		
**Inflammatory markers**							
C-reactive protein, mg/L, Median (IQR)	9.40 (3.70 - 16.80)	3.07 (0.93 - 6.46)	13.39 (8.20 - 19.32)	9.05 (4.00 - 15.70)	13.70 (5.10 - 22.65)	< 0.001	< 0.001
ESR, mm/hr, Median (IQR)	69.00 (42.00 - 97.00)	48.00 (31.00 - 78.50)	73.00 (50.50 - 97.00)	76.00 (50.75 - 104.25)	78.00 (41.00 - 105.00)	< 0.001	< 0.001
IL-6, pg/mL, Median (IQR)	19.00 (10.00 - 42.00)	12.00 (7.00 - 19.77)	17.00 (10.00 - 41.00)	21.00 (9.00 - 53.00)	31.00 (11.00 - 67.00)	< 0.001	0.54
Procalcitonin, ng/mL, Median (IQR)	0.20 (0.10 - 0.60)	0.10 (0.10 - 0.11)	0.20 (0.10 - 0.40)	0.40 (0.20 - 1.06)	0.50 (0.20 - 1.80)	< 0.001	0.001
Bands, %, Median (IQR)	2.00 (0.00 - 5.00)	2.00 (0.00 - 5.00)	2.00 (1.00 - 5.00)	2.00 (0.00 - 5.00)	2.00 (0.00 - 6.00)	0.86	0.06
LDH, U/L, Median (IQR)	377.00 (280.00 - 525.00)	275.00 (221.75 - 342.00)	448.00 (367.00 - 584.50)	353.50 (272.00 - 471.75)	514.00 (356.00 - 737.00)	< 0.001	< 0.001
Lymphocyte count, $\times 10^3$/uL, Median (IQR)	1.00 (0.70 - 1.43)	1.20 (0.90 - 1.70)	0.90 (0.60 - 1.20)	0.80 (0.50 - 1.10)	1.10 (0.70 - 1.61)	< 0.001	< 0.001
Neutrophil count, $\times 10^3$/uL, Median (IQR)	5.30 (3.70 - 7.90)	3.90 (2.90 - 5.20)	6.20 (4.50 - 8.40)	5.00 (3.40 - 7.00)	9.50 (6.70 - 13.05)	< 0.001	< 0.001
White blood cell count, $\times 10^3$/uL, Median (IQR)	7.20 (5.30 - 9.90)	5.80 (4.60 - 7.40)	7.80 (6.00 - 10.10)	6.60 (4.70 - 8.90)	12.10 (8.80 - 16.30)	< 0.001	< 0.001
**Inflammation & Hepatic markers**							< 0.001
Albumin, g/dL, Median (IQR)	3.70 (3.30 - 4.10)	4.00 (3.70 - 4.40)	3.70 (3.30 - 4.00)	3.50 (3.00 - 3.90)	3.40 (2.80 - 3.80)	< 0.001	< 0.001
Ferritin, ng/mL, Median (IQR)	645.00 (295.90 - 1347.00)	268.00 (129.00 - 470.50)	925.00 (530.25 - 1543.50)	713.70 (306.25 - 1599.42)	957.70 (376.00 - 1919.00)	< 0.001	< 0.001
**Hepatic markers**							
Alanine aminotransferase, U/L, Median (IQR)	29.00 (19.00 - 48.00)	23.00 (16.00 - 32.00)	44.00 (30.00 - 68.00)	20.00 (13.00 - 28.00)	36.00 (20.00 - 71.00)	< 0.001	< 0.001
Aspartate aminotransferase, U/L, Median (IQR)	39.00 (26.00 - 63.00)	28.00 (22.00 - 37.00)	56.00 (41.00 - 81.00)	32.00 (22.00 - 48.00)	56.00 (29.00 - 107.75)	< 0.001	< 0.001
Bilirubin, mg/dL, Median (IQR)	0.30 (0.20 - 0.60)	0.20 (0.20 - 0.40)	0.40 (0.20 - 0.70)	0.30 (0.20 - 0.50)	0.40 (0.20 - 0.80)	< 0.001	< 0.001
**Cardiovascular markers**							
Creatine kinase, U/L, Median (IQR)	154.00 (78.00 - 359.00)	109.00 (65.75 - 187.25)	207.50 (101.25 - 463.00)	141.00 (71.00 - 334.50)	226.00 (87.00 - 762.75)	< 0.001	< 0.001
Lactate, mmol/L, Median (IQR)	1.90 (1.40 - 2.60)	1.50 (1.20 - 2.00)	1.90 (1.50 - 2.60)	1.70 (1.30 - 2.40)	3.00 (2.00 - 4.50)	< 0.001	< 0.001
Troponin I, ng/mL, Median (IQR)	0.10 (0.06 - 0.30)	0.10 (0.00 - 0.10)	0.10 (0.04 - 0.20)	0.10 (0.10 - 0.27)	0.20 (0.10 - 0.60)	< 0.001	0.16
Troponin T, ng/mL, Median (IQR)	0.01 (0.01 - 0.03)	0.01 (0.01 - 0.01)	0.01 (0.01 - 0.01)	0.04 (0.01 - 0.10)	0.03 (0.01 - 0.12)	< 0.001	< 0.001
**Renal markers**							
Bicarbonate, mmol/L, Median (IQR)	23.00 (21.00 - 26.00)	24.00 (22.00 - 26.00)	24.00 (21.00 - 26.00)	22.00 (19.00 - 25.00)	21.00 (17.00 - 24.00)	< 0.001	< 0.001

BUN, mg/dL, Median (IQR)	17.00 (11.00 - 31.00)	13.00 (9.75 - 17.00)	16.00 (11.00 - 21.00)	39.00 (25.00 - 59.00)	38.00 (19.00 - 71.00)	< 0.001	< 0.001
Creatinine, mg/dL, Median (IQR)	1.00 (0.80 - 1.50)	0.87 (0.70 - 1.06)	0.95 (0.80 - 1.20)	2.20 (1.30 - 4.70)	1.57 (0.90 - 2.90)	< 0.001	< 0.001
Chloride, mmol/L, Median (IQR)	100.00 (97.00 - 104.00)	102.00 (99.00 - 104.00)	99.00 (96.00 - 102.00)	100.00 (95.00 - 105.00)	103.00 (96.00 - 110.00)	< 0.001	< 0.001
Sodium, mmol/L, Median (IQR)	137.00 (134.00 - 140.00)	138.00 (136.00 - 141.00)	135.00 (132.00 - 138.00)	137.00 (134.00 - 140.00)	139.00 (134.00 - 146.00)	< 0.001	< 0.001
**Hematologic markers**							
D-dimer, ng/mL, Median (IQR)	1360.00 (620.00 - 3370.00)	622.00 (370.00 - 1140.00)	1210.00 (640.00 - 2420.00)	1940.00 (960.00 - 3610.00)	3430.00 (1550.00 - 10300.00)	< 0.001	< 0.001
Hemoglobin, g/dL, Median (IQR)	13.10 (11.50 - 14.60)	13.20 (12.10 - 14.40)	13.90 (12.70 - 15.20)	10.80 (9.10 - 12.35)	12.80 (10.60 - 14.90)	< 0.001	< 0.001
Platelet count, $\times 10^3$/uL, Median (IQR)	211.00 (162.00 - 277.00)	206.00 (165.00 - 258.00)	212.00 (166.00 - 270.00)	191.00 (139.00 - 263.00)	257.00 (175.00 - 365.00)	< 0.001	< 0.001
Prothrombin time, s, Median (IQR)	13.30 (12.20 - 14.60)	12.60 (11.90 - 13.60)	13.40 (12.53 - 14.50)	13.20 (11.90 - 14.60)	14.30 (12.70 - 17.20)	< 0.001	< 0.001
Red blood cell distribution width, %, Median (IQR)	13.80 (12.90 - 15.00)	13.50 (12.80 - 14.50)	13.20 (12.60 - 14.10)	15.20 (13.90 - 16.90)	14.90 (13.60 - 17.00)	< 0.001	< 0.001
Glucose, mg/dL, Median (IQR)	121.00 (101.00 - 165.00)	107.00 (95.00 - 127.00)	127.00 (108.00 - 168.00)	127.00 (101.00 - 190.00)	153.00 (112.25 - 258.00)	< 0.001	< 0.001
**Other markers**							
Oxygen saturation, %, Median (IQR)	69.00 (50.00 - 85.00)	64.50 (47.50 - 84.90)	69.00 (51.00 - 85.00)	70.00 (47.00 - 80.50)	74.00 (56.00 - 92.00)	0.03	0.06
BMI, kg/m^2, Median (IQR)	28.00 (25.00 - 33.00)	29.00 (25.00 - 34.00)	29.00 (25.00 - 33.00)	27.00 (24.00 - 32.00)	26.00 (23.00 - 31.00)	< 0.001	0.51
**Comorbidity, (missing=590), N (%)**							
Hypertension	4744 (62.35)	1255 (51.16)	1480 (56.88)	1130 (86.00)	879 (70.89)	< 0.001	-
Diabetes	3104 (40.79)	701 (28.58)	978 (37.59)	802 (61.04)	623 (50.24)	< 0.001	-
Coronary artery disease	1753 (23.04)	379 (15.45)	446 (17.14)	571 (43.46)	357 (28.79)	< 0.001	-
Heart failure	1132 (14.88)	205 (8.36)	188 (7.23)	483 (36.76)	256 (20.65)	< 0.001	-
COPD	972 (12.77)	259 (10.56)	206 (7.92)	305 (23.21)	202 (16.29)	< 0.001	-
Asthma	1091 (14.34)	415 (16.92)	301 (11.57)	223 (16.97)	152 (12.26)	< 0.001	-
Cancer	1438 (18.90)	380 (15.49)	362 (13.91)	407 (30.97)	289 (23.31)	< 0.001	-
Hyperlipidemia	3262 (42.87)	851 (34.69)	1018 (39.12)	822 (62.56)	571 (46.05)	< 0.001	-
Obesity	3039 (37.07)	1076 (40.74)	1141 (40.65)	461 (33.04)	361 (26.62)	< 0.001	-
**Outcomes (60 days), N (%)**							
Mortality	1529 (18.65)	141 (5.34)	482 (17.17)	382 (27.38)	524 (38.64)	< 0.001	-
Mechanical ventilation (intubation	1154 (14.07)	129 (4.88)	495 (17.63)	212 (15.20)	318 (23.45)	< 0.001	-
ICU admission	1494 (18.22)	182 (6.89)	588 (20.95)	266 (19.07)	458 (33.78)	< 0.001	-
**Medications, N (%)**							
Antibiotics	2952 (36.00)	667 (25.26)	1117 (39.79)	577 (41.36)	591 (43.58)	< 0.001	-
Corticosteroids	1666 (20.32)	285 (10.79)	661 (23.55)	332 (23.80)	388 (28.61)	< 0.001	-
Enoxaparin	3312 (40.40)	968 (36.65)	1531 (54.54)	366 (26.24)	447 (32.96)	< 0.001	-
Heparin	1310 (15.98)	223 (8.44)	550 (19.59)	316 (22.65)	221 (16.30)	< 0.001	-
Vasopressor	608 (7.42)	90 (3.41)	291 (10.37)	92 (6.59)	135 (9.96)	< 0.001	-

Abbreviations: BUN = blood urea nitrogen; COPD = chronic obstructive pulmonary disease; ESR = Erythrocyte sedimentation rate; ICU = intensive care unit; IL-6 = Interleukin 6; IQR = Interquartile range; LDH = Lactate dehydrogenase.

[a] Comparisons across all 4 subphenotypes were performed using the Kruskal-Wallis test (with Dunn's test for post-hoc pairwise comparisons) or $\chi 2$ test.

[b] P-values, adjusting for age and sex, were calculated by analysis of covariance (ANCOVA) was performed based on General Linear Model.

Supplementary Table 8. Characteristics of the subphenotypes in the internal validation cohort.

Variable	Total	Subphenotype I	Subphenotype II	Subphenotype III	Subphenotype IV	P-value[a]	P-value (age and sex adjusted)[b]
No. of patients (%)	3519 (100)	800 (22.73)	1908 (54.22)	550 (15.63)	261 (7.42)	-	-
**Demographics**							
Age, y, Median (IQR)	63.51 (50.95 - 75.17)	58.53 (40.93 - 71.27)	62.12 (50.72 - 72.42)	70.82 (59.71 - 80.43)	73.97 (62.03 - 84.13)	< 0.001	-
Sex female, N (%)	1585 (45.04)	478 (59.75)	737 (38.63)	262 (47.64)	108 (41.38)	< 0.001	-
Race, N (%)							
White	838 (23.81)	206 (25.75)	464 (24.32)	122 (22.18)	46 (17.62)	< 0.001	-
Black	915 (26.00)	219 (27.38)	436 (22.85)	173 (31.45)	87 (33.33)		
Asian	193 (5.48)	40 (5.00)	111 (5.82)	26 (4.73)	16 (6.13)		
Other/unknown	1573 (44.70)	335 (41.87)	897 (47.01)	229 (41.63)	112 (42.91)		
**Inflammatory markers**							
C-reactive protein, mg/L, Median (IQR)	9.63 (4.39 - 16.42)	1.70 (0.50 - 5.13)	12.40 (7.43 - 18.50)	9.61 (4.198 - 16.43)	13.46 (6.45 - 20.55)	< 0.001	< 0.001
ESR, mm/hr, Median (IQR)	64.00 (41.00 - 93.00)	35.00 (18.50 - 59.00)	72.00 (53.00 - 95.00)	66.00 (46.00 - 103.00)	70.00 (47.50 - 100.25)	< 0.001	< 0.001
IL-6, pg/mL, Median (IQR)	16.50 (9.00 - 35.25)	10.00 (7.00 - 12.00)	17.00 (9.00 - 32.00)	16.50 (6.75 - 37.00)	81.50 (40.00 - 226.50)	< 0.001	< 0.001
Procalcitonin, ng/mL, Median (IQR)	0.20 (0.10 - 0.50)	0.10 (0.10 - 0.20)	0.20 (0.10 - 0.40)	0.30 (0.14 - 0.88)	0.81 (0.30 - 5.67)	< 0.001	< 0.001
Bands, %, Median (IQR)	2.00 (1.00 - 6.00)	3.00 (1.00 - 7.00)	2.00 (1.00 - 5.00)	2.00 (0.00 - 4.00)	3.00 (1.00 - 8.00)	0.08	0.15
LDH, U/L, Median (IQR)	382.00 (285.00 - 527.00)	241.00 (202.00 - 295.50)	410.00 (324.00 - 532.00)	376.00 (292.00 - 516.00)	630.00 (474.50 - 885.50)	< 0.001	< 0.001
Lymphocyte count, $\times 10^3$/uL, Median (IQR)	1.00 (0.70 - 1.40)	1.30 (0.90 - 1.90)	1.00 (0.70 - 1.30)	0.80 (0.50 - 1.10)	1.10 (0.80 - 1.70)	< 0.001	< 0.001
Neutrophil count, $\times 10^3$/uL, Median (IQR)	5.30 (3.70 - 7.70)	4.00 (2.70 - 5.70)	5.60 (4.00 - 7.90)	5.10 (3.70 - 7.20)	9.90 (7.20 - 12.93)	< 0.001	< 0.001
White blood cell count, $\times 10^3$/uL, Median (IQR)	7.10 (5.30 - 9.60)	6.10 (4.50 - 8.10)	7.30 (5.50 - 9.78)	6.80 (5.00 - 9.10)	12.10 (8.95 - 15.95)	< 0.001	< 0.001
**Inflammation & Hepatic markers**							
Albumin, g/dL, Median (IQR)	3.70 (3.30 - 4.10)	4.10 (3.70 - 4.40)	3.70 (3.30 - 4.10)	3.50 (3.00 - 3.80)	3.40 (2.90 - 3.80)	< 0.001	< 0.001
Ferritin, ng/mL, Median (IQR)	699.50 (297.00 - 1435.00)	223.00 (88.53 - 475.60)	854.15 (443.25 - 1544.72)	710.65 (261.00 - 1764.00)	1275.30 (617.00 - 2535.00)	< 0.001	< 0.001
**Hepatic markers**							
Alanine aminotransferase, U/L, Median (IQR)	30.00 (19.00 - 49.00)	20.00 (14.00 - 28.00)	38.00 (25.00 - 62.00)	24.00 (15.00 - 34.00)	40.00 (25.00 - 71.00)	< 0.001	< 0.001
Aspartate aminotransferase, U/L, Median (IQR)	40.00 (27.00 - 64.00)	25.00 (20.00 - 33.00)	49.00 (34.00 - 75.00)	37.00 (25.00 - 57.50)	64.50 (38.25 - 121.00)	< 0.001	< 0.001
Bilirubin, mg/dL, Median (IQR)	0.30 (0.20 - 0.60)	0.30 (0.20 - 0.50)	0.40 (0.20 - 0.60)	0.30 (0.20 - 0.50)	0.30 (0.20 - 0.60)	< 0.001	< 0.001
**Cardiovascular markers**							
Creatine kinase, U/L, Median (IQR)	151.00 (80.50 - 335.50)	100.50 (61.00 - 164.00)	171.00 (89.00 - 361.75)	135.00 (69.25 - 322.25)	405.00 (190.00 - 1174.00)	< 0.001	< 0.001
Lactate, mmol/L, Median (IQR)	1.90 (1.30 - 2.60)	1.45 (1.10 - 2.10)	1.90 (1.40 - 2.60)	1.80 (1.30 - 2.50)	3.40 (2.20 - 5.60)	< 0.001	< 0.001
Troponin I, ng/mL, Median (IQR)	0.10 (0.07 - 0.30)	0.07 (0.00 - 0.10)	0.10 (0.05 - 0.20)	0.10 (0.10 - 0.30)	0.27 (0.10 - 0.90)	< 0.001	0.09
Troponin T, ng/mL, Median (IQR)	0.01 (0.01 - 0.02)	0.01 (0.01 - 0.01)	0.01 (0.01 - 0.01)	0.04 (0.01 - 0.14)	0.07 (0.02 - 0.16)	< 0.001	< 0.001
**Renal markers**							
Bicarbonate, mmol/L, Median (IQR)	23.00 (21.00 - 26.00)	24.00 (22.00 - 26.00)	24.00 (21.00 - 26.00)	21.00 (19.00 - 25.00)	19.00 (16.00 - 23.00)	< 0.001	< 0.001
BUN, mg/dL, Median (IQR)	17.00 (11.00 - 29.00)	13.00 (10.00 - 19.00)	15.00 (11.00 - 22.00)	40.00 (23.00 - 62.50)	62.00 (36.00 - 95.00)	< 0.001	< 0.001

Creatinine, mg/dL, Median (IQR)	1.00 (0.80 - 1.50)	0.85 (0.70 - 1.10)	0.93 (0.80 - 1.20)	2.20 (1.20 - 4.80)	2.42 (1.51 - 4.00)	< 0.001	< 0.001
Chloride, mmol/L, Median (IQR)	100.00 (97.00 - 104.00)	103.00 (100.00 - 105.00)	99.00 (96.00 - 102.00)	101.00 (96.00 - 105.00)	110.00 (100.00 - 120.00)	< 0.001	< 0.001
Sodium, mmol/L, Median (IQR)	137.00 (134.00 - 140.00)	139.00 (137.00 - 141.00)	136.00 (133.00 - 139.00)	137.00 (134.00 - 141.00)	148.00 (139.00 - 159.00)	< 0.001	< 0.001
**Hematologic markers**							
D-dimer, ng/mL, Median (IQR)	1330.00 (585.25 - 3092.50)	636.00 (370.00 - 1450.00)	1210.00 (586.00 - 2740.00)	1975.00 (1008.00 - 3822.50)	4405.00 (2025.00 - 13922.50)	< 0.001	< 0.001
Hemoglobin, g/dL, Median (IQR)	13.30 (11.70 - 14.70)	13.10 (11.80 - 14.30)	13.90 (12.50 - 15.10)	10.40 (8.50 - 12.30)	13.50 (11.80 - 15.60)	< 0.001	< 0.001
Platelet count, $\times 10^3$/uL, Median (IQR)	210.00 (163.00 - 274.00)	212.00 (163.75 - 266.00)	209.00 (166.00 - 271.00)	205.00 (144.00 - 289.00)	221.50 (161.25 - 310.00)	0.14	0.002
Prothrombin time, s, Median (IQR)	13.20 (12.20 - 14.40)	12.40 (11.60 - 13.60)	13.40 (12.50 - 14.40)	13.15 (12.00 - 15.88)	14.10 (13.10 - 16.40)	< 0.001	< 0.001
Red blood cell distribution width, %, Median (IQR)	13.70 (13.00 - 14.90)	13.70 (13.00 - 14.70)	13.30 (12.70 - 14.20)	15.50 (14.10 - 17.80)	14.70 (13.80 - 16.17)	< 0.001	< 0.001
Glucose, mg/dL, Median (IQR)	122.00 (101.50 - 164.00)	103.00 (91.75 - 120.00)	125.00 (107.00 - 169.00)	133.00 (100.00 - 192.00)	162.00 (126.00 - 258.00)	< 0.001	< 0.001
**Other markers**							
Oxygen saturation, %, Median (IQR)	72.00 (48.50 - 90.00)	68.00 (47.00 - 82.75)	72.00 (48.00 - 86.00)	76.00 (49.50 - 93.00)	91.00 (67.00 - 95.50)	0.06	0.11
BMI, kg/m^2, Median (IQR)	28.00 (24.00 - 33.00)	28.00 (24.00 - 33.00)	29.00 (25.00 - 33.00)	27.00 (23.00 - 31.25)	26.00 (22.00 - 30.00)	< 0.001	< 0.001
**Comorbidity, (missing=590), N (%)**							
Hypertension	2051 (62.86)	371 (51.60)	1053 (58.99)	428 (84.75)	199 (78.35)	< 0.001	-
Diabetes	1288 (39.47)	187 (26.01)	674 (37.76)	302 (59.80)	125 (49.21)	< 0.001	-
Coronary artery disease	774 (23.72)	134 (18.64)	320 (17.93)	234 (46.34)	86 (33.86)	< 0.001	-
Heart failure	488 (14.96)	76 (10.57)	185 (10.36)	174 (34.46)	53 (20.87)	< 0.001	-
COPD	385 (11.80)	83 (11.54)	153 (8.57)	108 (21.39)	41 (16.14)	< 0.001	-
Asthma	463 (14.19)	138 (19.19)	208 (11.65)	88 (17.43)	29 (11.42)	< 0.001	-
Cancer	575 (17.62)	125 (17.39)	243 (13.61)	156 (30.89)	51 (20.08)	< 0.001	-
Hyperlipidemia	1364 (41.80)	236 (32.82)	689 (38.60)	303 (60.00)	136 (53.54)	< 0.001	-
Obesity	1261 (35.83)	282 (35.25)	743 (38.94)	171 (31.09)	65 (24.90)	< 0.001	-
**Outcomes (60 days), N (%)**							
Mortality	696 (19.78)	37 (4.62)	343 (17.98)	173 (31.45)	143 (54.79)	< 0.001	-
Mechanical ventilation (intubation	497 (14.12)	22 (2.75)	289 (15.15)	110 (20.00)	76 (29.12)	< 0.001	-
ICU admission	661 (18.78)	49 (6.12)	382 (20.02)	130 (23.64)	100 (38.31)	< 0.001	-
**Medications, N (%)**							
Antibiotics	1234 (35.07)	183 (22.88)	734 (38.47)	186 (33.82)	131 (50.19)	< 0.001	-
Corticosteroids	733 (20.83)	80 (10.00)	431 (22.59)	127 (23.09)	95 (36.40)	< 0.001	-
Enoxaparin	1403 (39.87)	242 (30.25)	947 (49.63)	141 (25.64)	73 (27.97)	< 0.001	-
Heparin	552 (15.69)	73 (9.12)	316 (16.56)	118 (21.45)	45 (17.24)	< 0.001	-
Vasopressor	276 (7.84)	22 (2.75)	166 (8.70)	54 (9.82)	34 (13.03)	< 0.001	-

Abbreviations: BUN = blood urea nitrogen; COPD = chronic obstructive pulmonary disease; ESR = Erythrocyte sedimentation rate; ICU = intensive care unit; IL-6 = Interleukin 6; IQR = Interquartile range; LDH = Lactate dehydrogenase.

[a] Comparisons across all 4 subphenotypes were performed using the Kruskal-Wallis test (with Dunn's test for post-hoc pairwise comparisons) or $\chi 2$ test.

[b] P-values, adjusting for age and sex, were calculated by analysis of covariance (ANCOVA) was performed based on General Linear Model.

Supplementary Table 9. Characteristics of the predicted subphenotypes in the external validation cohort.

Variable	Total	Subphenotype I	Subphenotype II	Subphenotype III	Subphenotype IV	P-value[a]	P-value (age and sex adjusted)[b]
No. of patients (%)	2700 (100)	918 (34.00)	929 (34.41)	499 (18.48)	354 (13.11)	-	-
**Demographics**							
Age, y, Median (IQR)	65.58 (51.08 - 77.39)	58.52 (39.53 - 71.23)	65.03 (53.78 - 74.69)	69.44 (57.23 - 80.55)	76.53 (68.07 - 85.33)	< 0.001	-
Sex female, N (%)	1305 (48.33)	544 (59.26)	346 (37.24)	267 (53.51)	148 (41.81)	< 0.001	-
Race, N (%)							
White	675 (25.00)	239 (26.03)	238 (25.62)	124 (24.85)	74 (20.90)	< 0.001	-
Black	545 (20.19)	186 (20.26)	136 (14.64)	135 (27.05)	88 (24.86)		
Asian	28 (1.04)	7 (0.76)	16 (1.72)	3 (0.60)	2 (0.56)		
Other/unknown	1452 (53.78)	486 (52.94)	539 (58.02)	237 (47.50)	190 (53.67)		
**Inflammatory markers**							
C-reactive protein, mg/L, Median (IQR)	11.30 (5.50 - 20.15)	6.16 (1.47 - 11.21)	15.35 (8.99 - 23.99)	9.78 (4.46 - 18.33)	16.29 (8.99 - 27.69)	< 0.001	< 0.001
ESR, mm/hr, Median (IQR)	72.00 (50.00 - 97.00)	56.00 (36.50 - 81.00)	74.00 (56.00 - 97.00)	80.00 (55.00 - 103.00)	86.00 (60.00 - 109.00)	< 0.001	< 0.001
IL-6, pg/mL, Median (IQR)	45.20 (11.15 - 108.40)	10.50 (5.00 - 38.60)	67.25 (22.95 - 128.93)	40.30 (18.80 - 97.50)	134.60 (59.42 - 303.15)	< 0.001	< 0.001
Procalcitonin, ng/mL, Median (IQR)	0.20 (0.10 - 0.60)	0.10 (0.10 - 0.20)	0.30 (0.10 - 0.50)	0.30 (0.20 - 1.30)	0.90 (0.40 - 2.85)	< 0.001	< 0.001
Bands, %, Median (IQR)	3.00 (2.00 - 5.00)	2.00 (1.00 - 4.00)	3.00 (1.00 - 5.00)	3.00 (2.00 - 5.25)	4.00 (2.00 - 6.50)	0.02	0.05
LDH, U/L, Median (IQR)	419.00 (310.50 - 599.50)	307.00 (232.00 - 397.50)	497.00 (381.00 - 689.00)	365.50 (291.25 - 475.50)	621.00 (455.00 - 873.00)	< 0.001	< 0.001
Lymphocyte count, $\times 10^3$/uL, Median (IQR)	1.00 (0.70 - 1.50)	1.10 (0.80 - 1.60)	1.00 (0.80 - 1.40)	0.80 (0.60 - 1.20)	1.00 (0.60 - 1.50)	< 0.001	0.01
Neutrophil count, $\times 10^3$/uL, Median (IQR)	5.40 (3.70 - 8.10)	4.10 (3.00 - 5.40)	7.00 (4.90 - 9.95)	4.50 (3.20 - 6.20)	8.90 (6.50 - 12.75)	< 0.001	< 0.001
White blood cell count, $\times 10^3$/uL, Median (IQR)	7.30 (5.40 - 10.20)	6.20 (4.80 - 7.70)	8.90 (6.60 - 12.30)	6.00 (4.60 - 8.00)	10.95 (8.10 - 15.65)	< 0.001	< 0.001
**Inflammation & Hepatic markers**							
Albumin, g/dL, Median (IQR)	3.80 (3.40 - 4.10)	4.00 (3.70 - 4.40)	3.70 (3.40 - 4.10)	3.60 (3.20 - 3.92)	3.40 (3.00 - 3.70)	< 0.001	< 0.001
Ferritin, ng/mL, Median (IQR)	672.65 (333.03 - 1276.00)	346.70 (169.60 - 655.50)	894.20 (480.60 - 1475.25)	637.90 (260.00 - 1331.00)	979.10 (531.45 - 1962.75)	< 0.001	< 0.001
**Hepatic markers**							
Alanine aminotransferase, U/L, Median (IQR)	27.00 (17.00 - 47.00)	23.00 (16.00 - 33.00)	40.00 (23.00 - 67.00)	18.00 (13.00 - 26.00)	39.00 (23.00 - 73.00)	< 0.001	< 0.001
Aspartate aminotransferase, U/L, Median (IQR)	39.00 (26.00 - 65.00)	30.00 (22.00 - 42.00)	55.00 (35.00 - 87.00)	30.00 (21.00 - 42.00)	72.00 (43.00 - 134.00)	< 0.001	< 0.001
Bilirubin, mg/dL, Median (IQR)	0.30 (0.20 - 0.50)	0.20 (0.10 - 0.40)	0.40 (0.20 - 0.60)	0.20 (0.10 - 0.40)	0.40 (0.20 - 0.70)	< 0.001	< 0.001
**Cardiovascular markers**							
Creatine kinase, U/L, Median (IQR)	154.50 (79.00 - 347.75)	103.00 (61.00 - 208.00)	166.00 (92.00 - 393.75)	131.00 (66.50 - 243.00)	364.00 (135.00 - 1023.00)	< 0.001	< 0.001
Lactate, mmol/L, Median (IQR)	1.60 (1.20 - 2.30)	1.20 (1.00 - 1.60)	1.70 (1.30 - 2.40)	1.30 (1.00 - 1.80)	2.90 (2.00 - 4.60)	< 0.001	< 0.001
Troponin I, ng/mL, Median (IQR)	0.00 (0.00 - 0.05)	0.00 (0.00 - 0.00)	0.00 (0.00 - 0.00)	0.00 (0.00 - 0.00)	0.10 (0.10 - 0.20)	< 0.001	0.29
Troponin T, ng/mL, Median (IQR)	0.02 (0.01 - 0.04)	0.01 (0.01 - 0.01)	0.01 (0.01 - 0.03)	0.04 (0.02 - 0.10)	0.08 (0.04 - 0.15)	< 0.001	< 0.001
**Renal markers**							
Bicarbonate, mmol/L, Median (IQR)	23.00 (20.00 - 25.00)	24.00 (22.00 - 26.00)	23.00 (20.00 - 25.00)	22.00 (19.00 - 25.00)	19.00 (16.00 - 22.00)	< 0.001	< 0.001
BUN, mg/dL, Median (IQR)	18.00 (12.00 - 33.00)	12.00 (9.00 - 16.00)	17.00 (12.00 - 26.00)	35.00 (22.00 - 57.00)	54.00 (34.00 - 83.00)	< 0.001	< 0.001

Creatinine, mg/dL, Median (IQR)	1.10 (0.80 - 1.60)	0.90 (0.70 - 1.10)	1.00 (0.80 - 1.30)	2.10 (1.20 - 4.90)	2.10 (1.30 - 3.50)	< 0.001	< 0.001
Chloride, mmol/L, Median (IQR)	100.00 (96.00 - 103.00)	100.00 (98.00 - 103.00)	98.00 (95.00 - 101.00)	101.00 (96.00 - 104.00)	105.00 (98.00 - 114.00)	< 0.001	< 0.001
Sodium, mmol/L, Median (IQR)	138.00 (135.00 - 141.00)	138.00 (136.00 - 141.00)	137.00 (134.00 - 139.00)	138.00 (135.00 - 141.00)	143.00 (137.00 - 153.50)	< 0.001	< 0.001
**Hematologic markers**							
D-dimer, ng/mL, Median (IQR)	1400.00 (700.00 - 3100.00)	750.00 (400.00 - 1400.00)	1500.00 (900.00 - 2800.00)	1900.00 (1100.00 - 3400.00)	4250.00 (2325.00 - 13875.00)	< 0.001	< 0.001
Hemoglobin, g/dL, Median (IQR)	13.00 (11.40 - 14.30)	13.20 (12.10 - 14.30)	13.70 (12.50 - 14.80)	10.60 (9.30 - 11.90)	12.70 (10.80 - 14.45)	< 0.001	< 0.001
Platelet count, $\times 10^3$/uL, Median (IQR)	203.00 (157.00 - 262.50)	198.00 (158.00 - 248.00)	216.00 (170.00 - 290.00)	179.00 (139.00 - 240.00)	210.00 (159.00 - 289.00)	< 0.001	< 0.001
Prothrombin time, s, Median (IQR)	14.20 (13.40 - 15.30)	13.60 (13.10 - 14.30)	14.10 (13.40 - 15.00)	14.45 (13.60 - 15.57)	16.10 (14.80 - 19.20)	< 0.001	< 0.001
Red blood cell distribution width, %, Median (IQR)	13.90 (13.00 - 15.00)	13.50 (12.80 - 14.30)	13.40 (12.80 - 14.30)	15.30 (14.00 - 17.20)	15.00 (13.90 - 16.40)	< 0.001	< 0.001
Glucose, mg/dL, Median (IQR)	125.00 (104.00 - 176.00)	108.00 (96.00 - 126.00)	138.00 (115.00 - 219.75)	122.00 (100.25 - 156.00)	183.00 (127.00 - 288.00)	< 0.001	< 0.001
**Other markers**							
Oxygen saturation, %, Median (IQR)	97.10 (92.00 - 98.70)	95.25 (73.88 - 98.45)	96.80 (93.00 - 98.70)	97.00 (92.40 - 98.80)	98.00 (91.60 - 98.82)	0.70	0.23
BMI, kg/m^2, Median (IQR)	28.00 (24.00 - 33.00)	29.00 (25.00 - 34.00)	28.00 (25.00 - 32.00)	26.50 (23.00 - 32.00)	26.00 (22.00 - 30.00)	< 0.001	0.65
**Comorbidity, (missing=590), N (%)**							
Hypertension	539 (20.01)	131 (14.35)	181 (19.48)	151 (30.32)	76 (21.47)	< 0.001	-
Diabetes	438 (16.26)	82 (8.98)	169 (18.19)	121 (24.30)	66 (18.64)	< 0.001	-
Coronary artery disease	167 (6.20)	26 (2.85)	50 (5.38)	64 (12.85)	27 (7.63)	< 0.001	-
Heart failure	153 (5.68)	18 (1.97)	39 (4.20)	60 (12.05)	36 (10.17)	< 0.001	-
COPD	74 (2.75)	17 (1.86)	28 (3.01)	18 (3.61)	11 (3.11)	0.21	-
Asthma	101 (3.75)	51 (5.59)	31 (3.34)	14 (2.81)	5 (1.41)	0.001	-
Cancer	175 (6.50)	51 (5.59)	55 (5.92)	45 (9.04)	24 (6.78)	0.07	-
Hyperlipidemia	193 (7.16)	42 (4.60)	72 (7.75)	49 (9.84)	30 (8.47)	0.001	-
Obesity	910 (33.70)	339 (36.93)	327 (35.20)	160 (32.06)	84 (23.73)	< 0.001	-
**Outcomes (60 days), N (%)**							
Mortality	556 (20.59)	56 (6.10)	204 (21.96)	112 (22.44)	184 (51.98)	< 0.001	-
Mechanical ventilation (intubation	248 (9.19)	38 (4.14)	113 (12.16)	40 (8.02)	57 (16.10)	< 0.001	-
ICU admission	-	-	-	-	-	-	-
**Medications, N (%)**							
Antibiotics	1739 (64.41)	450 (49.02)	655 (70.51)	351 (70.34)	283 (79.94)	< 0.001	-
Corticosteroids	741 (27.44)	176 (19.17)	318 (34.23)	161 (32.26)	86 (24.29)	< 0.001	-
Enoxaparin	1788 (66.22)	604 (65.80)	744 (80.09)	271 (54.31)	169 (47.74)	< 0.001	-
Heparin	978 (36.22)	188 (20.48)	305 (32.83)	291 (58.32)	194 (54.80)	< 0.001	-
Vasopressor	219 (8.11)	23 (2.51)	103 (11.09)	41 (8.22)	52 (14.69)	< 0.001	-

Abbreviations: BUN = blood urea nitrogen; COPD = chronic obstructive pulmonary disease; ESR = Erythrocyte sedimentation rate; ICU = intensive care unit; IL-6 = Interleukin 6; IQR = Interquartile range; LDH = Lactate dehydrogenase.

[a] Comparisons across all 4 subphenotypes were performed using the Kruskal-Wallis test (with Dunn's test for post-hoc pairwise comparisons) or $\chi 2$ test.

[b] P-values, adjusting for age and sex, were calculated by analysis of covariance (ANCOVA) was performed based on General Linear Model.

Supplementary Table 10. Characteristics of the subphenotypes identified in the leave-one-center-out procedure among all studied patients.

Variable	Total	Subphenotype I	Subphenotype II	Subphenotype III	Subphenotype IV	P-value[a]	P-value (age and sex adjusted)[b]
No. of patients (%)	14418 (100)	5319 (36.89)	4627 (32.09)	3454 (23.96)	1018 (7.06)	-	-
**Demographics**							
Age, y, Median (IQR)	64.01 (50.72 - 75.55)	59.36 (43.89 - 71.73)	62.41 (51.48 - 72.59)	71.66 (58.82 - 81.78)	68.57 (58.91 - 77.87)	< 0.001	-
Sex female, N (%)	6677 (46.31)	2849 (53.56)	1741 (37.63)	1719 (49.77)	368 (36.15)	< 0.001	-
Race, N (%)							
White	3549 (24.62)	1384 (26.02)	1117 (24.14)	833 (24.12)	215 (21.12)	< 0.001	-
Black	3615 (25.07)	1324 (24.89)	885 (19.13)	1104 (31.96)	302 (29.67)		
Asian	630 (4.37)	191 (3.59)	279 (6.03)	119 (3.45)	41 (4.03)		
Other/unknown	6624 (45.95)	2420 (52.94)	2346 (50.7)	1398 (40.48)	460 (45.19)		
**Inflammatory markers**							
C-reactive protein, mg/L, Median (IQR)	9.90 (4.10 - 174.00)	4.93 (1.32 - 10.50)	13.42 (7.60 - 20.44)	10.30 (4.60 - 17.70)	16.80 (9.39 - 26.24)	< 0.001	< 0.001
ESR, mm/hr, Median (IQR)	69.00 (45.00 - 96.25)	55.00 (34.00 - 80.00)	75.00 (54.00 - 99.00)	76.00 (48.75 - 104.00)	82.00 (53.00 - 106.00)	< 0.001	< 0.001
IL-6, pg/mL, Median (IQR)	22.00 (10.00 - 57.83)	13.00 (6.00 - 29.00)	21.00 (11.00 - 55.00)	33.00 (13.00 - 80.10)	60.50 (20.75 - 156.18)	< 0.001	0.002
Procalcitonin, ng/mL, Median (IQR)	0.20 (0.10 - 0.60)	0.10 (0.10 - 0.20)	0.20 (0.10 - 0.46)	0.40 (0.20 - 1.30)	0.70 (0.30 - 2.30)	< 0.001	< 0.001
Bands, %, Median (IQR)	2.00 (1.00 - 5.00)	3.00 (1.00 - 6.00)	2.00 (0.00 - 5.00)	2.30 (0.30 - 6.00)	3.00 (1.00 - 6.00)	0.02	0.01
LDH, U/L, Median (IQR)	390.00 (288.00 - 542.00)	299.00 (232.00 - 391.50)	443.00 (350.00 - 585.25)	402.00 (301.00 - 562.50)	645.00 (429.50 - 908.50)	< 0.001	< 0.001
Lymphocyte count, $\times 10^3$/uL, Median (IQR)	1.00 (0.70 - 1.40)	1.10 (0.80 - 1.60)	1.00 (0.70 - 1.30)	1.00 (0.60 - 1.40)	0.90 (0.60 - 1.30)	< 0.001	0.01
Neutrophil count, $\times 10^3$/uL, Median (IQR)	5.40 (3.70 - 7.90)	4.00 (2.90 - 5.40)	6.40 (4.70 - 8.90)	6.00 (4.10 - 8.80)	9.20 (6.20 - 13.10)	< 0.001	< 0.001
White blood cell count, $\times 10^3$/uL, Median (IQR)	7.20 (5.30 - 9.90)	5.80 (4.50 - 7.50)	8.20 (6.30 - 10.70)	7.80 (5.70 - 11.00)	11.30 (7.80 - 15.70)	< 0.001	< 0.001
**Inflammation & Hepatic markers**							
Albumin, g/dL, Median (IQR)	3.70 (3.30 - 4.10)	4.00 (3.60 - 4.30)	3.60 (3.20 - 4.00)	3.50 (3.00 - 3.90)	3.40 (3.00 - 3.80)	< 0.001	< 0.001
Ferritin, ng/mL, Median (IQR)	661.00 (303.00 - 1352.25)	372.00 (172.00 - 772.02)	846.75 (454.00 - 1506.50)	722.25 (289.50 - 1536.00)	1265.00 (612.92 - 2463.50)	< 0.001	< 0.001
**Hepatic markers**							
Alanine aminotransferase, U/L, Median (IQR)	29.00 (18.00 - 48.00)	25.00 (17.00 - 38.00)	39.00 (25.00 - 64.00)	23.00 (15.00 - 37.00)	39.00 (22.00 - 83.00)	< 0.001	< 0.001
Aspartate aminotransferase, U/L, Median (IQR)	40.00 (26.00 - 64.00)	32.00 (23.00 - 47.00)	50.00 (34.00 - 75.00)	37.00 (24.00 - 61.00)	67.50 (36.00 - 146.25)	< 0.001	< 0.001
Bilirubin, mg/dL, Median (IQR)	0.30 (0.20 - 0.60)	0.30 (0.20 - 0.50)	0.40 (0.20 - 0.60)	0.30 (0.20 - 0.50)	0.40 (0.20 - 0.80)	< 0.001	< 0.001
**Cardiovascular markers**							
Creatine kinase, U/L, Median (IQR)	153.00 (79.00 - 349.75)	124.00 (72.00 - 235.00)	159.00 (83.25 - 358.75)	176.00 (77.00 - 493.25)	293.00 (115.00 - 890.75)	< 0.001	< 0.001
Lactate, mmol/L, Median (IQR)	1.80 (1.30 - 2.60)	1.50 (1.10 - 2.10)	1.90 (1.50 - 2.60)	1.90 (1.30 - 2.80)	3.20 (2.10 - 5.00)	< 0.001	< 0.001
Troponin I, ng/mL, Median (IQR)	0.10 (0.04 - 0.27)	0.10 (0.00 - 0.10)	0.10 (0.03 - 0.20)	0.12 (0.10 - 0.37)	0.30 (0.10 - 1.10)	< 0.001	< 0.001
Troponin T, ng/mL, Median (IQR)	0.01 (0.01 - 0.03)	0.01 (0.01 - 0.01)	0.01 (0.01 - 0.01)	0.05 (0.02 - 0.14)	0.03 (0.01 - 0.10)	< 0.001	< 0.001
**Renal markers**							
Bicarbonate, mmol/L, Median (IQR)	23.00 (21.00 - 26.00)	24.00 (22.00 - 26.00)	24.00 (21.00 - 26.00)	22.00 (19.00 - 25.00)	19.00 (16.00 - 22.00)	< 0.001	< 0.001

BUN, mg/dL, Median (IQR)	17.00 (11.00 - 31.00)	13.00 (10.00 - 18.00)	15.00 (11.00 - 22.00)	41.00 (22.00 - 69.00)	39.00 (25.00 - 62.50)	< 0.001	< 0.001
Creatinine, mg/dL, Median (IQR)	1.00 (0.80 - 1.50)	0.90 (0.70 - 1.10)	0.92 (0.80 - 1.20)	2.06 (1.10 - 4.46)	1.75 (1.20 - 2.80)	< 0.001	< 0.001
Chloride, mmol/L, Median (IQR)	100.00 (97.00 - 104.00)	101.00 (98.00 - 104.00)	98.00 (95.00 - 101.00)	103.00 (98.00 - 109.00)	99.00 (95.00 - 104.00)	< 0.001	< 0.001
Sodium, mmol/L, Median (IQR)	137.00 (134.00 - 140.00)	138.00 (136.00 - 140.00)	135.00 (132.00 - 138.00)	139.00 (136.00 - 144.00)	137.00 (133.00 - 141.00)	< 0.001	< 0.001
**Hematologic markers**							
D-dimer, ng/mL, Median (IQR)	1370.00 (630.00 - 3250.00)	770.00 (410.00 - 1500.00)	1300.00 (660.00 - 2710.00)	2530.00 (1110.25 - 5843.00)	3740.00 (1920.00 - 11840.00)	< 0.001	< 0.001
Hemoglobin, g/dL, Median (IQR)	13.10 (11.50 - 14.60)	13.40 (12.12 - 14.70)	13.80 (12.60 - 15.00)	11.20 (9.50 - 13.00)	13.10 (11.10 - 14.90)	< 0.001	< 0.001
Platelet count, $\times 10^3$/uL, Median (IQR)	209.00 (162.00 - 273.00)	196.00 (156.00 - 247.00)	226.00 (175.00 - 298.00)	206.00 (153.00 - 278.00)	228.00 (169.00 - 308.00)	< 0.001	< 0.001
Prothrombin time, s, Median (IQR)	13.60 (12.60 - 14.80)	13.20 (12.30 - 14.00)	13.60 (12.70 - 14.70)	14.20 (12.70 - 16.10)	14.60 (13.20 - 16.80)	< 0.001	< 0.001
Red blood cell distribution width, %, Median (IQR)	13.80 (13.00 - 15.00)	13.45 (12.80 - 14.40)	13.30 (12.70 - 14.20)	15.30 (14.10 - 17.20)	14.30 (13.30 - 15.90)	< 0.001	< 0.001
Glucose, mg/dL, Median (IQR)	122.00 (102.00 - 166.00)	108.00 (96.00 - 127.00)	135.00 (111.00 - 202.00)	126.00 (101.00 - 172.00)	213.00 (141.00 - 354.00)	< 0.001	< 0.001
**Other markers**							
Oxygen saturation, %, Median (IQR)	78.00 (54.00 - 93.60)	70.00 (49.00 - 90.00)	74.00 (54.00 - 92.00)	80.00 (55.00 - 95.15)	91.00 (70.00 - 98.00)	< 0.001	< 0.001
BMI, kg/m^2, Median (IQR)	28.00 (24.00 - 33.00)	29.00 (25.00 - 33.00)	28.00 (25.00 - 33.00)	27.00 (23.00 - 32.00)	27.00 (24.00 - 32.00)	< 0.001	0.59
**Comorbidity, (missing=590), N (%)**							
Hypertension	7334 (54.06)	2284 (45.29)	2230 (52.01)	2194 (67.32)	626 (64.14)	< 0.001	-
Diabetes	4830 (35.60)	1198 (23.76)	1664 (38.81)	1432 (43.94)	536 (54.92)	< 0.001	-
Coronary artery disease	2694 (19.86)	695 (13.78)	643 (15.00)	1101 (33.78)	255 (26.13)	< 0.001	-
Heart failure	1773 (13.07)	425 (8.43)	325 (7.58)	841 (25.81)	182 (18.65)	< 0.001	-
COPD	1431 (10.55)	464 (9.20)	311 (7.25)	536 (16.45)	120 (12.30)	< 0.001	-
Asthma	1655 (12.20)	708 (14.04)	461 (10.75)	390 (11.97)	96 (9.84)	< 0.001	-
Cancer	2188 (16.13)	722 (14.32)	553 (12.90)	748 (22.95)	165 (16.91)	< 0.001	-
Hyperlipidemia	4819 (35.52)	1451 (28.77)	1481 (34.54)	1476 (45.29)	411 (42.11)	< 0.001	-
Obesity	5210 (36.14)	2070 (38.91)	1718 (37.13)	1071 (31.01)	351 (34.48)	< 0.001	-
**Outcomes (60 days), N (%)**							
Mortality	2781 (19.29)	488 (9.17)	764 (16.51)	1036 (29.99)	493 (48.43)	< 0.001	-
Mechanical ventilation (intubation	1899 (13.17)	374 (7.03)	716 (15.47)	503 (14.56)	306 (30.06)	< 0.001	-
ICU admission	2155 (14.95)	424 (7.97)	830 (17.94)	586 (16.97)	315 (30.94)	<0.001	-
**Medications, N (%)**							
Antibiotics	5925 (41.09)	1816 (34.14)	1966 (42.49)	1529 (44.27)	614 (60.31)	< 0.001	-
Corticosteroids	3140 (21.78)	866 (16.28)	1116 (24.12)	813 (23.54)	345 (33.89)	< 0.001	-
Enoxaparin	6503 (45.10)	2399 (45.10)	2541 (54.92)	1154 (33.41)	409 (40.18)	< 0.001	-
Heparin	2840 (19.70)	756 (14.21)	899 (19.43)	891 (25.80)	294 (28.88)	< 0.001	-
Vasopressor	1103 (7.65)	256 (4.81)	431 (9.31)	273 (7.90)	143 (14.05)	< 0.001	-

Abbreviations: BUN = blood urea nitrogen; COPD = chronic obstructive pulmonary disease; ESR = Erythrocyte sedimentation rate; ICU = intensive care unit; IL-6 = Interleukin 6; IQR = Interquartile range; LDH = Lactate dehydrogenase.

[a] Comparisons across all 4 subphenotypes were performed using the Kruskal-Wallis test (with Dunn's test for post-hoc pairwise comparisons) or $\chi 2$ test.

[b] P-values, adjusting for age and sex, were calculated by analysis of covariance (ANCOVA) was performed based on General Linear Model.

Supplementary Table 11. Distributions of SDoH variables by subphenotypes in the development cohort

SDoH variables	Total	Subphenotype I	Subphenotype II	Subphenotype III	Subphenotype IV	P-value[a]
# of patients	8199 (100)	2707 (33.02)	3047 (37.16)	1486 (18.12)	959 (11.70)	-
Median household income, $, Mean (IQR)	524090 (37015 - 71225)	52052 (37015 - 71437)	52409 (40733 - 71477)	52052 (37243 - 69799)	51477 (37015 - 63652)	0.006
Crowding housing, %, Mean (IQR)	9.30 (6.00 - 14.48)	9.30 (6.00 - 14.10)	9.80 (6.40 - 15.70)	9.30 (6.00 - 13.50)	9.80 (6.40 - 15.90)	0.01
No high school degree, %, Mean (IQR)	20.49 (14.16 - 27.53)	20.49 (14.16 - 27.53)	20.49 (14.16 - 27.53)	20.49 (14.16 - 26.11)	20.49 (15.75 - 26.10)	0.34
Essential occupation rate, %, Mean (IQR)	44.12 (37.42 - 47.57)	43.87 (37.32 - 47.57)	43.87 (37.32 - 47.45)	45.28 (37.42 - 47.57)	46.30 (39.65 - 47.57)	<0.001
Non-white resident rate, %, Mean (IQR)	72.32 (42.69 - 84.25)	72.32 (41.90 - 84.64)	69.67 (41.95 - 82.27)	76.81 (47.29 - 84.64)	77.38 (51.80 - 84.25)	<0.001
Unemployed rate, %, Mean (IQR)	7.80 (5.70 - 10.05)	7.80 (5.70 - 10.05)	7.20 (5.50 - 10.10)	7.80 (5.80 - 10.30)	8.80 (6.50 - 10.70)	<0.001

Abbreviations: IQR = Interquartile range; LDH = Lactate dehydrogenase; SDoH = social determinants of health.
[a] Comparisons across all 4 subphenotypes were performed using the Kruskal-Wallis test (with Dunn's test for post-hoc pairwise comparisons) or $\chi 2$ test.

Supplementary Table 12. Log odds ratio by logistic regression analyses showing associations between SDoH variables and 60-day mortality risk in each subphenotype in the development cohort

SDoH variables	Subphenotype I		Subphenotype II		Subphenotype III		Subphenotype IV	
	Log odds (SD)	P-value	Log odds (SD)	P-value	Log odds (SD)	P-value	Log odds (SD)	P-value
Logistic regression analysis adjusting for age and sex.								
Median household income	-0.09 (0.08)	0.28	-0.06 (0.05)	0.20	-0.13 (0.07)	0.06	-0.14 (0.08)	0.07
Crowding housing	0.05 (0.08)	0.51	0.09 (0.04)	0.05	0.07 (0.07)	0.30	0.11 (0.08)	0.12
No high school degree	0.09 (0.08)	0.27	0.11 (0.05)	0.03	0.13 (0.06)	0.05	0.14 (0.07)	0.06
Essential occupation rate	0.07 (0.08)	0.40	0.12 (0.05)	0.02	0.18 (0.07)	0.01	0.15 (0.07)	0.04
Non-white resident rate	0.02 (0.08)	0.77	0.09 (0.05)	0.10	0.07 (0.06)	0.29	0.18 (0.07)	0.01
Unemployed rate	-0.04 (0.08)	0.63	0.03 (0.05)	0.53	0.11 (0.07)	0.10	0.19 (0.07)	0.01
Logistic regression analysis adjusting for age, sex, and clinical variables								
Median household income	-0.06 (0.09)	0.51	0.01 (0.06)	0.76	-0.13 (0.08)	0.10	-0.03 (0.09)	0.73
Crowding housing	-0.01 (0.09)	0.87	0.03 (0.05)	0.55	0.08 (0.07)	0.28	0.02 (0.08)	0.77
No high school degree	0.05 (0.09)	0.57	0.04 (0.05)	0.43	0.13 (0.07)	0.07	0.05 (0.08)	0.53
Essential occupation rate	0.08 (0.09)	0.39	0.08 (0.06)	0.15	0.16 (0.08)	0.04	0.08 (0.09)	0.34
Non-white resident rate	-0.025 (0.09)	0.79	0.07 (0.06)	0.22	0.05 (0.08)	0.51	0.13 (0.09)	0.13
Unemployed rate	-0.11 (0.10)	0.26	-0.04 (0.06)	0.51	0.06 (0.07)	0.28	0.10 (0.08)	0.25

Logistic regression analyses were performed the associations between each SDoH variable with 60-day mortality risk in each subphenotype, adjusting for age and sex or for age, sex, and clinical variables.
Abbreviations: SD = standard deviation.

Supplementary Table 13. Hazard ratio by Cox regression analyses showing associations between SDoH variables and 60-day mortality risk in each subphenotype in the development cohort

SDoH variables	Subphenotype I		Subphenotype II		Subphenotype III		Subphenotype IV	
	HR (95% CI)	P-value	HR (95% CI)	P-value	HR (95% CI)	P-value	HR (95% CI)	P-value
Median household income	1.02 (0.89 - 1.16)	0.83	0.97 (0.89 - 1.06)	0.53	0.92 (0.82 - 1.03)	0.13	0.97 (0.87 - 1.08)	0.59
Crowding housing	1.01 (0.87 - 1.17)	0.89	1.09 (1.00 - 1.19)	0.04	1.06 (0.95 - 1.18)	0.31	1.05 (0.95 - 1.15)	0.33
No high school degree	1.01 (0.88 - 1.17)	0.85	1.08 (1.00 - 1.18)	0.06	1.09 (0.98 - 1.21)	0.10	1.06 (0.95 - 1.17)	0.06
Essential occupation rate	1.02 (0.88 - 1.17)	0.80	1.08 (0.99 - 1.18)	0.07	1.12 (1.00 - 1.25)	0.04	1.07 (0.97 - 1.19)	0.20
Non-white resident rate	0.96 (0.84 - 1.11)	0.59	1.06 (0.97 - 1.15)	0.21	1.03 (0.92 - 1.14)	0.62	1.08 (0.98 - 1.20)	0.11
Unemployed rate	0.88 (0.75 - 1.02)	0.09	0.98 (0.90 - 1.07)	0.61	1.06 (0.95 - 1.18)	0.27	1.09 (0.99 - 1.20)	0.07

Cox regression analyses were performed the associations between each SDoH variable with 60-day mortality risk in each subphenotype, adjusting for age and sex.
Abbreviations: HR = Hazard ratio; CI = confidence interval.

Supplementary Table 14. Characteristics of the identified SDoH strata in the development cohort

Variable	Total	Stratum H	Stratum M	Stratum L	P-value[a]	P-value (age and sex adjusted)[b]
No. of patients, N (%)	7862 (100)[c]	1933 (24.59)	3912 (49.76)	2017 (25.66)	-	
**Demographics**						
Age, Median (IQR)	63.58 (50.51 - 75.16)	64.76 (50.70 - 76.33)	63.99 (51.34 - 75.33)	62.10 (49.11 - 73.83)	< 0.001	< 0.001
Sex female, N (%)	3649 (46.41)	867 (44.85)	1848 (47.24)	934 (46.31)	0.22	-
**COVID confirmation date**						
Time of COVID confirmation since March 1st, 2020, Day Median (IQR)	35 (27 - 47)	35 (26 - 47)	35 (28 - 46)	36 (28 - 49)	0.001	0.005
**Neighborhood conditions**						
Median household income, $, Mean (IQR)	52409.00 (37015.00 - 71225.00)	89466.00 (72314.00 - 117069.00)	52409.00 (50108.00 - 63652.00)	36046.00 (28277.00 - 37015.00)	< 0.001	< 0.001
Crowding housing, %, Mean (IQR)	9.30 (6.00 - 14.48)	3.70 (2.10 - 7.70)	9.30 (6.50 - 10.90)	15.90 (15.90 - 18.10)	< 0.001	< 0.001
No high school degree, %, Mean (IQR)	20.49 (14.16 - 27.53)	8.90 (5.42 - 13.85)	20.49 (18.86 - 24.34)	34.24 (26.10 - 37.57)	< 0.001	< 0.001
Essential occupation rate, %, Mean (IQR)	44.12 (37.42 - 47.57)	32.34 (27.77 - 37.19)	44.24 (40.70 - 47.57)	47.45 (46.50 - 52.67)	< 0.001	< 0.001
Non-white resident rate, %, Mean (IQR)	72.32 (42.69 - 84.25)	26.82 (22.03 - 35.93)	76.81 (58.45 - 84.70)	82.27 (77.38 - 86.81)	< 0.001	< 0.001
Unemployed rate, %, Mean (IQR)	7.80 (5.70 - 10.50)	4.90 (4.00 - 5.90)	8.20 (6.80 - 9.40)	12.10 (10.70 - 12.50)	< 0.001	< 0.001
**60-day outcomes**						
Mortality	1522 (19.36)	340 (17.59)	779 (19.91)	403 (19.98)	0.08	-
Mechanical ventilation (intubation	1148 (14.60)	298 (15.42)	556 (14.21)	294 (14.58)	0.47	-
ICU admission	1459 (18.56)	416 (21.52)	717 (18.33)	326 (16.16)	< 0.001	-

Abbreviations: IQR = Interquartile range; LDH = Lactate dehydrogenase; SDoH = social determinants of health.

[a] Comparisons across all 4 subphenotypes were performed using the Kruskal-Wallis test (with Dunn's test for post-hoc pairwise comparisons) or χ2 test.

[b] P-values, adjusting for age and sex, were calculated by analysis of covariance (ANCOVA) was performed based on General Linear Model.

[c] Of 8199 patients in the development cohort, 7862 patients containing SDoH data were used to derive SDoH strata.

Supplementary Table 15. Distributions of SDoH strata by subphenotypes in the development cohort

SDoH strata	Total	Subphenotype I	Subphenotype II	Subphenotype III	Subphenotype IV	P-value[a]
# of patients	7862	2593	2905	1432	932	-
Stratum H	1933 (24.59%)	690 (26.61%)	732 (25.20%)	331 (23.11%)	180 (19.31%)	< 0.001
Stratum M	3912 (49.76%)	1230 (47.44%)	1441 (49.60%)	759 (53.00%)	482 (51.72%)	
Stratum L	2017 (25.66%)	673 (25.95%)	732 (25.20%)	342 (23.88%)	270 (28.97%)	

Abbreviations: SDoH = social determinants of health.
[a] Comparisons across all 4 subphenotypes were performed using $\chi 2$ test.

Supplementary Table 16. Logistic regression analyses and Cox regression analyses showing associations between SDoH strata and 60-day mortality risk by subphenotypes in the development cohort

Subphenotypes	Log odds (SD)			Hazard ratio (95% CI)		
	SDoH stratum H[a]	SDoH stratum M	SDoH stratum L	SDoH stratum H[a]	SDoH stratum M	SDoH stratum L
Subphenotype I	-	0.18 (-0.19 - 0.56)	0.13 (-0.31 - 0.58)	-	1.04 (0.74 - 1.47)	0.99 (0.66 - 1.49)
Subphenotype II	-	0.05 (-0.18 - 0.30)	0.17 (-0.10 - 0.45)	-	1.09 (0.89 - 1.35)	1.13 (0.89 - 1.44)
Subphenotype III	-	0.31 (0 - 0.64)	0.23 (-0.15 - 0.60)	-	1.26 (0.96 - 1.66)	1.14 (0.82 - 1.57)
Subphenotype IV	-	0.15 (-0.20 - 0.50)	0.40 (0.02 - 0.79)*	-	1.05 (0.82 - 1.35)	1.20 (0.92 - 1.57)

Abbreviations: CI = confidence interval; SD = standard deviation; SDoH = social determinants of health
[a] Reference group.
* P-value < 0.05
