# Supplementary material for: Clinical subphenotypes in COVID-19: derivation, validation, prediction, temporal patterns, and interaction with social determinants of health
Source: NPJ Digit Med. 2021 Jul 14;4:110. doi: 10.1038/s41746-021-00481-w (PMC8280198; doi:10.1038/s41746-021-00481-w)
Supplement: Supplementary file 2 — Reporting Summary [file 41746_2021_481_MOESM2_ESM.pdf]

nature research

Corresponding author(s): Rainu Kaushal, Edward J. Schenck, Fei Wang

Last updated by author(s): Jun 11, 2021

Reporting Summary

Nature Research wishes to improve the reproducibility of the work that we publish. This form provides structure for consistency and transparency in reporting. For further information on Nature Research policies, see our Editorial Policies and the Editorial Policy Checklist.

Statistics

For all statistical analyses, confirm that the following items are present in the figure legend, table legend, main text, or Methods section.

n/a	Confirmed	
☐	☒	The exact sample size (*n*) for each experimental group/condition, given as a discrete number and unit of measurement
☐	☒	A statement on whether measurements were taken from distinct samples or whether the same sample was measured repeatedly
☐	☒	The statistical test(s) used AND whether they are one- or two-sided<br>*Only common tests should be described solely by name; describe more complex techniques in the Methods section.*
☐	☒	A description of all covariates tested
☐	☒	A description of any assumptions or corrections, such as tests of normality and adjustment for multiple comparisons
☐	☒	A full description of the statistical parameters including central tendency (e.g. means) or other basic estimates (e.g. regression coefficient) AND variation (e.g. standard deviation) or associated estimates of uncertainty (e.g. confidence intervals)
☒	☐	For null hypothesis testing, the test statistic (e.g. *F*, *t*, *r*) with confidence intervals, effect sizes, degrees of freedom and *P* value noted<br>*Give P values as exact values whenever suitable.*
☒	☐	For Bayesian analysis, information on the choice of priors and Markov chain Monte Carlo settings
☒	☐	For hierarchical and complex designs, identification of the appropriate level for tests and full reporting of outcomes
☒	☐	Estimates of effect sizes (e.g. Cohen's *d*, Pearson's *r*), indicating how they were calculated

Our web collection on statistics for biologists contains articles on many of the points above.

Software and code

Policy information about availability of computer code

Data collection	Data collection was performed by independent members of INSIGHT clinical research network. The clinical data was provided in CSV files.
Data analysis	All computer codes in this study are available at https://github.com/ChangSu10/COVID-Insight-subphenotyping. Implementation of our work is based on Python 3.7 and R 3.6. More specifically, clustering models were implemented based on Python packages ‘scikit-learn 0.23.2’ (https://scikit-learn.org/stable/) and ‘scipy 1.5.3’ (https://www.scipy.org). Supervised predictive modeling was based on ‘XGBoost 1.2.1’ (https://xgboost.readthedocs.io/en/latest/) and ‘SHAP 0.35.0’ (https://shap.readthedocs.io/en/latest/). Data dimension reduction and visualization were performed based on Python package ‘UMAP-learn 0.3.9’ (https://umap-learn.readthedocs.io/en/latest/). R package ‘NbClust’ (https://cran.r-project.org/web/packages/NbClust/NbClust.pdf) was used to calculate measures of clusters to determine the optimal cluster number in agglomerative hierarchical clustering. Chord diagrams were created using R package ‘circlize’ (https://cran.r-project.org/web/packages/circlize/index.html). All statistical tests and survival analyses were performed based on R.

For manuscripts utilizing custom algorithms or software that are central to the research but not yet described in published literature, software must be made available to editors and reviewers. We strongly encourage code deposition in a community repository (e.g. GitHub). See the Nature Research guidelines for submitting code & software for further information.

Data

Policy information about availability of data

All manuscripts must include a data availability statement. This statement should provide the following information, where applicable:

- Accession codes, unique identifiers, or web links for publicly available datasets
- A list of figures that have associated raw data
- A description of any restrictions on data availability

All data studied in this work can be downloaded from INSIGHT clinical research network at https://insightcrn.org/our-data/, via request.

Field-specific reporting

Please select the one below that is the best fit for your research. If you are not sure, read the appropriate sections before making your selection.

☒ Life sciences ☐ Behavioural & social sciences ☐ Ecological, evolutionary & environmental sciences

For a reference copy of the document with all sections, see nature.com/documents/nr-reporting-summary-flat.pdf

Life sciences study design

All studies must disclose on these points even when the disclosure is negative.

Sample size	A total of 14418 patients with confirmed COVID-19 between March 1st and June 12th 2020 were included for analysis from the five major medical centers in New York City (NYC), including New York University Langone Medical Center (NYU-LMC), New York Presbyterian - Weill Cornell Medical Center (NYP-WCMC), Mount Sinai Health System (MSHS), Montefiore Medical Center (MMC), and New York Presbyterian - Columbia University Medical Center (NYP-CUMC).
Data exclusions	Exclusion criteria include younger than 18 years old; duplicated patient IDs; having no emergency department (ED) or inpatient (IP) admission within 14 days after COVID-19 confirmation; or having missing values on all clinical variables.
Replication	We originally derived subphenotypes using the development cohort based on agglomerative hierarchical clustering. In order to evaluate the reproducibility, we validated our subphenotypes in three ways. First, we performed sensitivity analyses using the development cohort. Second, we used the internal validation cohort and re-derived subphenotypes using the same agglomerative hierarchical clustering with the primary analysis for validation. Third, for the aims of confirming subphenotypes and their usability, we trained predictive model of subphenotypes in the development cohort and used it to predict subphenotypes in an external validation cohort. Last, to assess stability of the subphenotypes across the five medical centers, we further performed leave-one-center-out analysis.
Randomization	we combined patients of four centers and randomly divided them into the development cohort (70%) and internal validation cohort (30%). Patients of the last center were used as the external validation cohort. In the leave-one-center-out analysis, each center was used as the within-loop validation cohort and the remaining four were used as the development cohort.
Blinding	This is not relevant to our study because the research team was not involved with the data collection process.

Reporting for specific materials, systems and methods

We require information from authors about some types of materials, experimental systems and methods used in many studies. Here, indicate whether each material, system or method listed is relevant to your study. If you are not sure if a list item applies to your research, read the appropriate section before selecting a response.

Materials & experimental systems

n/a	Involved in the study	
☒	☐	Antibodies
☒	☐	Eukaryotic cell lines
☒	☐	Palaeontology and archaeology
☒	☐	Animals and other organisms
☒	☐	Human research participants
☐	☒	Clinical data
☒	☐	Dual use research of concern

Methods

n/a	Involved in the study	
☒	☐	ChIP-seq
☒	☐	Flow cytometry
☒	☐	MRI-based neuroimaging

Clinical data

Policy information about clinical studies

All manuscripts should comply with the ICMJE guidelines for publication of clinical research and a completed CONSORT checklist must be included with all submissions.

Clinical trial registration	NA
Study protocol	The derivation and validation of subphenotypes, statistical analysis the subphenotype characterization, and studies of impact of socioeconomics factors on biological subphenotypes are described in Methods section in the manuscript and eMethods section in Supplement. We use only existing observational EHR data and we do not recruit new patients or collect new clinical variables from participants as part of this study.
Data collection	We used data of COVID-19 patients from INSIGHT Clinical Research Network (CRN). INSIGHT is funded by the Patient-Centered Outcomes Research Institute (PCORI) and aggregates clinical data of diverse patient populations across five academic medical centers in New York City (NYC), including New York University Langone Medical Center (NYU-LMC), New York Presbyterian - Weill Cornell Medical Center (NYP-WCMC), New York Presbyterian - Columbia University Medical Center (NYP-CUMC), Mount Sinai Health System (MSHS), and Montefiore Medical Center (MMC). Patients with confirmed COVID-19 between March 1st and June 12th 2020 were included for analysis from the five centers. we combined patients of four centers and randomly divided them into the development cohort (70%) and internal validation cohort (30%). Patients of the last center were used as the external validation cohort. Lab test data were used for subphenotype identification. Patient demographics, lab test data, comorbidity, and outcome data were used to characterize the identified subphenotypes. To explore the impact of SDoH status on the subphenotypes, we extracted patients' neighborhood socioeconomic characteristics from the 2018 American Community Survey.
Outcomes	NA
